# Cognitive aging and verbal labeling in continuous visual memory

**DOI:** 10.3758/s13421-020-01043-3

**Published:** 2020-05-29

**Authors:** Alicia Forsberg, Wendy Johnson, Robert H. Logie

**Affiliations:** 1 The University of Edinburgh, Edinburgh, UK; 2 Department of Psychological Sciences, The University of Missouri, University of Missouri-Columbia, 210 McAlester Hall, Columbia, MO 65211-2500 USA

**Keywords:** Visual working memory, Cognitive aging, Delayed estimation, Verbal labeling, Memory precision

## Abstract

The decline of working memory (WM) is a common feature of general cognitive decline, and visual and verbal WM capacity appear to decline at different rates with age. Visual material may be remembered via verbal codes or visual traces, or both. Souza and Skóra, *Cognition*, *166*, 277–297 (2017) found that labeling boosted memory in younger adults by activating categorical visual long-term memory (LTM) knowledge. Here, we replicated this and tested whether it held in healthy older adults. We compared performance in silence, under instructed overt labeling (participants were asked to say color names out loud), and articulatory suppression (repeating irrelevant syllables to prevent labeling) in the delayed estimation paradigm. Overt labeling improved memory performance in both age groups. However, comparing the effect of overt labeling and suppression on the number of coarse, categorical representations in the two age groups suggested that older adults used verbal labels subvocally more than younger adults, when performing the task in silence. Older adults also appeared to benefit from labels differently than younger adults. In younger adults labeling appeared to improve visual, continuous memory, suggesting that labels activated visual LTM representations. However, for older adults, labels did not appear to enhance visual, continuous representations, but instead boosted memory via additional verbal (categorical) memory traces. These results challenged the assumption that visual memory paradigms measure the same cognitive ability in younger and older adults, and highlighted the importance of controlling differences in age-related strategic preferences in visual memory tasks.

**Electronic supplementary material:**

The online version of this article (10.3758/s13421-020-01043-3) contains supplementary material, which is available to authorized users.

Visual working memory (WM)—maintaining visual information in memory during a short interval when it is no longer present but needed for an upcoming task—declines steeply with age (Babcock & Salthouse, 1990; Bowles & Salthouse, 2003; Craik, Luo, & Sakuta, 2010; Gazzaley, Cooney, Rissman, & D’Esposito, 2005; Johnson, Logie, & Brockmole, 2010; Jost, Bryck, Vogel, & Mayr; 2010; Park et al., 2002; Reuter-Lorenz & Sylvester, 2005). This age-related decline has practical importance, as WM is believed to underpin effective operation of other cognitive functions, such as perception and problem solving (e.g., Ma, Husain, & Bays, 2014), and to be related to general intelligence (e.g., Unsworth, Fukuda, Awh, & Vogel, 2014) and reasoning ability (e.g., Conway, Kane, & Engle, 2003; Kyllonen & Christal, 1990). However, there is disagreement about whether WM is best conceptualized as a unitary construct (e.g., Cowan, 2005; Oberauer, 2013) or as consisting of different components. For instance, the multicomponent model of WM (Baddeley, 1986, 2012; Baddeley & Logie, 1999; Logie, 2011) is based on the postulate that visuospatial and verbal information are stored separately in dedicated storage buffers, which may also rely on separate rehearsal mechanisms (Baddeley, 2012; Logie, 2011), possibly supported by different brain networks (Gruber, 2001; Jonides et al., 1996; but see also D’Esposito & Postle, 2015). Furthermore, visual WM capacity appears to decline at a faster rate with age than verbal WM capacity (Johnson et al., 2010), supporting the notion that they do not rely on the same mechanisms. In this paper, we investigated whether younger and older adults use verbal labels for retaining visually presented stimuli to the same extent, and whether such labels have the same effect on memory in the different age groups.

The nature of the relation between language and cognition has been debated for decades (Hunt & Angoli, 1991; Watson, 1924; Whorf, 1956). Verbalization—translating visual perceptual input into phonologically based verbal code—has a central role in this debate. Although translating visual input into verbal labels is well established as a default—perhaps sometimes even unavoidable—tendency (Conrad, 1964; Postle, D’Esposito, & Corkin, 2005; Postle & Hamidi, 2006; Shulman 1971; Simons, 1996), its impact on cognition is unclear (Lewis-Peacock, Drysdale, & Postle, 2014). For instance, verbalization might be detrimental to cognitive tasks such as decision-making (Wilson and Schooler, 1991), analogical reasoning (Lane & Schooler, 2004) and visual imagery (Brandimonte, Hitch, & Bishop, 1992). This well-established phenomenon is known as *verbal overshadowing,* because resources are thought to be allocated to the verbal label at the expense of the original task (Schooler & Engstler-Schooler, 1990), but the mechanisms behind it are still disputed (Chin & Schooler, 2008; Hatano, Ueno, Kitagami, & Kawaguchi, 2015).

Indeed, many memoranda—in everyday life as well as in memory experiments—may be remembered via verbal codes or visual traces, or both, among other possibilities. For example, if you put down your drink at a party and an identical glass filled with something else appeared next to it, marking your own drink in your mind could be achieved using a verbal description (‘the yellow one’), as well as a visual representation of the yellowness of the drink in it. The visual memory representation would help you grab your champagne instead of what would be someone else’s orange juice. Neuroscience evidence supports the notion that individual participants generate visual, phonological and semantic mental codes when viewing visual stimuli (Lewis-Peacock et al., 2014). Despite this tendency to translate visual representations into verbal codes, visual and verbal WM are typically measured separately, and a given task is assumed to measure one or the other, despite evidence that both verbal and visual codes might be stored for visually presented material (e.g., Logie, 2018; Logie, Saito, Morita, Varma, & Norris, 2016; Paivio, 1971; Saito, Logie, Morita, & Law, 2008). In the WM literature, there is little doubt that perceptual input results in different types of mental codes—both within and between individuals—which may interfere with one another in complex ways (e.g., Morey & Cowan, 2004). Moreover, while domain-specific stores are not emphasized in unitary conceptions of WM, they are not explicitly rejected (Cowan, 2005; Cowan, Saults, & Blume, 2014; Oberauer, 2013). It is generally agreed that subvocal rehearsal of verbal material is a separate mechanism that can support memory; however, the existence of a visuospatial rehearsal mechanism (Logie, 1995) is more contentious (Morey & Mall, 2012; Morey & Miron, 2016). Hence, the limits of visual WM and its decline with age are often investigated while attempting to prevent verbal labeling using concurrent articulatory suppression (i.e., repeating nonsense syllables out loud during the encoding and/or retention period; Allen, Baddeley, & Hitch, 2006; Hollingworth & Rasmussen, 2010; Logie, Brockmole, & Vandenbroucke, 2009; Matsukura & Hollingworth, 2011; van Lamsweerde & Beck, 2012), or assumed to be prevented by presenting items very briefly.

The past decade has seen a new way to measure visual WM. *Delayed estimation paradigms* provide precise, continuous measures of memory, in line with the idea that WM resources are allocated among items in memory and remembering more items leads to loss of precision as resources are spread more thinly (Ma et al., 2014). In these paradigms, participants reproduce features in memory on a continuous report scale (Prinzmetal, Amiri, Allen, & Edwards, 1998; Wilken & Ma, 2004; Zhang & Luck, 2008), which enables analysis of the distribution of the magnitudes of recall errors. These can be characterized by mathematical models that estimate both WM precision and the proportions of items participants remember (Wilken & Ma, 2004; Zhang & Luck, 2008). For example, participants recall colors by selecting among different color shades arranged around a color-wheel continuum after a brief delay (e.g., Bays, Catalao, & Husain, 2009; Bays, Wu, & Husain, 2011; Emrich & Ferber, 2012; Fougnie & Alvarez, 2011; Fougnie, Asplund, & Marois, 2010; Fougnie, Suchow, & Alvarez, 2012; Peich, Husain, & Bays, 2013; van den Berg, Shin, Chou, George, & Ma, 2012; Wilken & Ma, 2004; Zhang & Luck, 2008, 2009, 2011). Crucially, this paradigm differs from traditional memory tasks where the to-be-remembered items belong to limited sets of categories (e.g., ‘red’, ‘blue’) and rough categorical retention alone is sufficient to perform the task perfectly (e.g., Allen et al., 2006; Cocchini, Logie, Della Sala, MacPherson, & Baddeley, 2002; Kane et al., 2004; Saults & Cowan, 2007). Initially, researchers measuring color memory precision assumed that all colors were stored as visual, continuous representations (Zhang & Luck, 2008). Others later questioned this assumption. Several studies indicated that even continuous color values were stored in WM based on categorization, as participants’ responses clustered closely around specific, prototypical color values instead of being evenly distributed along the color-wheel continuum (Bae, Olkkonen, Allred, Wilson, & Flombaum, 2014; Bae, Olkkonen, Allred, & Flombaum, 2015; Donkin, Nosofsky, Gold, & Shiffrin, 2015; Hardman, Vergauwe, & Ricker, 2017; Olsson & Poom, 2005; Souza & Skóra, 2017).

Hardman et al. (2017) found that their model that included a mechanism for remembering rough categories (e.g., ‘purple’) outperformed the continuous representation-only model. Others have partially addressed what produces such categorical responding. Categorization of colors is not necessarily verbal—it occurs in perceptual tasks (Bae et al., 2015) and when labeling is unlikely (see Bae et al., 2014). However, Bae et al. (2015) observed that color values remembered with higher precision within and across participants were shades most commonly selected as prototypical (e.g., the ‘bluest shade of blue’) by an independent sample of participants. Based on such findings, assigning and subvocally rehearsing a label (e.g., ‘blue’) was proposed as a likely mechanism for remembering colors categorically (Donkin et al., 2015). To investigate this, Souza and Skóra (2017) manipulated participants’ use of verbal labels in the color-wheel paradigm. They used Hardman et al.’s (2017) model to separate continuous and categorical responses, and found that labeling increased both the number of categorical and continuous representations and the precision with which continuous representations were remembered in healthy young adults. Hence, verbal labels did not appear to boost memory representations by merely adding verbal memory representations. Instead, Souza and Skóra suggested that labeling boosted memory by activating categorical visual long-term memory (LTM) knowledge, which enabled participants to rely on two visual representations: a temporary visual representation of what the color looked like (independent of labeling), and a representation of the given visual category in LTM. As most research using the color-wheel paradigm has been done using younger adult samples and there is evidence that older adults may perform better in tasks which allow verbal rehearsal of labels (e.g., Johnson et al., 2010), we investigated how verbal labeling impacted visual memory in healthy older adults.

Older adults might rely more on verbal labels in visual tasks to support or compensate for declining visual memory (Baltes & Baltes, 1990; Park & Reuter-Lorenz, 2009; Reuter-Lorenz & Park, 2014), thus relying on a different cognitive ability than younger adults to perform the same task. This would be in line with literature suggesting that older adults show more severe deficits for visuospatial than verbal material (see Jenkins, Myerson, Joerding, & Hale, 2000; see also Bopp & Verhaeghen, 2005; Johnson et al., 2010; Leonards, Ibanez, & Giannakopoulos, 2002; Logie & Maylor, 2009; Myerson, Hale, Rhee, & Jenkins, 1999; Park et al., 2002, for a meta-analysis regarding age-deficits in verbal memory). Johnson et al. (2010) found evidence supporting the notion that younger and older adults may rely on different cognitive abilities, investigating the factor structures of performance on various WM tasks in 95,000 online participants of different ages. They found that for older participants, visual memory seemed more related to some general cognitive capacity, but in younger participants, it seemed to reflect a more specific capacity. However, in older people, verbal memory appeared related to a more general factor, supporting the idea that older people might rely on their verbal memory ability to perform a visual memory task. Additionally, Reuter-Lorenz et al. (2000) observed lateral organization of the WM system in young participants, whereas older participants showed considerable activity in both left and right frontal sites for both verbal and visual WM, suggesting that younger and older adults may engage different brain areas to different extents when performing the same task (Reuter-Lorenz, 2002).

Merely testing the simple hypothesis that older adults are worse at various cognitive tasks than younger adults (i.e., the ‘dull hypothesis’; Logie, Horne, & Petit, 2015; Perfect & Maylor, 2000) arguably does little to further our understanding of how or why cognition declines with age. Instead, identifying and investigating different subprocesses which decline at different rates and how this might drive older adults to recruit relatively spared cognitive functions to compensate for cognitive functions that decline with age is likely a more informative approach to understanding what changes in healthy aging. If visual tasks allow verbal labeling of stimuli, and younger and older adults differ in the extent to which they rely on such labeling, problematic confounds likely occur—especially if visual WM paradigms used to measure a given phenomenon differ in the extent to which verbalization is possible. For instance, age-related differences in verbal recoding could be problematic in paradigms measuring visual feature-binding if single features lend themselves to efficient verbal labeling and rehearsal and bound objects do not—while ‘red, blue, green’ may be feasible to verbalize during a typical memory retention interval, ‘red-circle, blue-square, green-triangle’ would likely be much more cumbersome (Brockmole & Logie, 2013; see Forsberg, Johnson, & Logie, 2019, for a summary of feature-binding paradigms and the role of verbal labeling, but see also Sense, Morey, Prince, Heathcote, & Morey, 2016). Indeed, age-related binding deficits in delayed estimation tasks were observed in some experimental settings (memory for color and orientation of bars; Peich et al., 2013), but not others (locations of complex, hard-to-name fractal objects; Pertzov, Heider, Liang, & Husain, 2015). Furthermore, if older adults favor verbal, categorical representations over continuous ones, this could cause reduced precision in older adults (Peich et al., 2013), since on average, categorical responses may be further from the correct shades than responses based on precise visual representations. Differential reliance on verbal labeling in participants of different age groups would likely go unnoticed in the majority of visual WM paradigms. Here, we tested whether older adults support a declining visual WM system by relying on verbal rehearsal of labels using a relatively intact phonological loop system (Baddeley & Hitch, 2018) by manipulating verbal labeling in the delayed estimation paradigm. We conducted a conceptual replication of Souza and Skóra (2017, Experiment 4), including both younger and older participants. Like them, we used a mixture model to distinguish rough categorical (‘verbal’) representations from continuous (‘visual’) representations, based on knowledge of the specific shade (Hardman et al., 2017). This model relies on the assumption that correct responses in this color memory task belong to one of these two types of representations: continuous representations, which are visual in nature (as they are based on a precise representation of what the specific color looked like), and categorical representations, which are not necessarily visual (as they tend to cluster around prototypical color values, and could theoretically be maintained without a visual representation (e.g., only remembering the verbal label ‘red’). Hardman et al. (2017) found that when they included a mechanism for remembering rough categories (e.g., ‘purple’), the model outperformed the continuous representation-only model. However, we did not assume that continuous representations as classified by the model correspond *perfectly* to visual memory traces, and categorical to verbal. Indeed, the link between verbal labeling and the two types of representations is an empirical question, which we explore with the explicit labeling manipulations below.

We investigated the following three questions:

1. Do older adults spontaneously use verbal labels more than younger adults when free to do so? We compared the proportion of categorical vs. continuous responses by the two age groups performing the task in silence, with instructed labeling, or under suppression. If participants generally subvocally label, the number of continuous versus categorical representations should be similar to that during instructed labeling. If they do not spontaneously label, it should be similar to that under suppression.
2. Does preventing labeling and/or rehearsal of labels impair older adults’ memory performance more than it impairs younger adults’ performance? If older adults’ ‘visual’ memory performance (as measured by the visual delayed estimation task) depends on verbal labeling (i.e., a verbal strategy) to compensate for poor visual memory, their memory, overall, should be more impaired while verbal labeling is disrupted by articulatory suppression, compared with younger adults.
3. Do older adults benefit from labels in the same way as younger adults? This can be tested by comparing instructed labeling with suppression (i.e., disrupted labeling) in the two age groups. Souza and Skóra (2017) found that, in younger adults, labeling was associated with increased categorical and continuous memory representations, and improved precision, consistent with the labeling benefit being due to activated visual LTM representations. However, if verbal labels overwrite continuous representations in older adults, instructed labeling would result in fewer continuous representations, but more categorical representations, which would be consistent with the verbal recoding hypothesis (see Schooler & Engstler-Schooler, 1990). Alternatively, labeling might be beneficial because it adds a verbal (categorical) representation to the visual WM trace (the dual-trace [visual + verbal] hypothesis). Then, the number of continuous (‘visual’) representations would be the same, but there would be additional, categorical (‘verbal’) representations. Evidence suggesting that older adults benefit from having two traces (Osaka, Otsuka, & Osaka, 2012) might support this hypothesis. If one of these alternative hypotheses better explains the labeling benefit in older adults, this would indicate that being allowed to label impacts performance via separate processes in younger and older adults*.*

Researchers comparing color memory precision in younger and older adults typically assume that the same cognitive ability (visuospatial WM) is measured in all participants. With our three research questions, we explicitly tested this assumption. The method and analyses were preregistered via the Open Science Framework (osf.io/m64px).

We examined whether preventing or instructing verbal labeling would affect various aspects of participants’ memory performance in the different age groups differently. Specifically, we distinguished continuous and categorical responding (Hardman, 2017) and used an explicit labeling paradigm to separate performance under instructed overt labeling, articulatory suppression to prevent labeling, and in silence (similar to Souza & Skóra, 2017) to test the following three hypotheses.

- H_1_*: Do younger and older adults differ in the probability of storage in WM (of the to-be-remembered colors)?* If participants in one age group depend more on verbal labels for their visual WM performance, *preventing labels* should impair their memory performance comparatively more than it does for the other age group.
- H_2_: *Do younger and older adults differ in the probability that the representation in memory is continuous as opposed to categorical*? If participants in one age group spontaneously use verbal labels more in silence, their relative performance in silence to that in the two verbalization manipulations (labeling, suppression) should differ from that of the other age group. Also, if participants in different age groups benefit differently from verbal labels, labeling (compared with suppression) may result in increases in different types of representations (i.e., differential gains in continuous and/or categorical representations).
- H_3_: *Do younger and older adults differ in the imprecision of the continuous representation in memory?* Applying a continuous/categorical model to delayed recall data from older adults is useful to test the possibility that differential favoring of categorical representations over continuous may contribute to differences in precision between younger and older adults (Peich et al., 2013), since, on average, categorical responses are further from the correct shades than are responses based on precise visual representations (i.e., continuous representations). Here, we investigated continuous precision in the age groups without categorical representations using Hardman’s (2017) model to separate these types of responses.

## Methods

### Participants

To reach our target sample size of 60 participants,^1^ we recruited 32 younger adults and 33 older adults, and excluded and replaced two younger and three older adults for not completing all trials, per our preregistered exclusion criteria. The final sample consisted of 30 younger adults (18–27 years old, *M* = 22.0, *SD* = 2.7, nine males), and 30 older adults (62–78 years, *M* = 68.6, *SD* = 4.9, 10 males). All participants reported having normal or corrected-to-normal vision and were without color-vision deficits (indicated by less than five errors on the on-screen version of the Dvorine pseudo-isochromatic plates; Dvorine, 1963). All older adults scored above the recommended cutoff point for cognitive impairments of 25 on the ACE-III-Mini (Hsieh et al., 2015; *M* = 28.5, *SD* = 1.5), completed after the color memory task. Younger (*M* = 16.07, *SD* = 2.07) and older adults (*M* = 16.22, *SD* = 4.07) did not differ in how many years of full-time education they had completed, *t*(58) = −.18, *p* = .856, *d* = −0.047. Younger adults received £7.50 for their participation, and older adults £10, as their sessions differed in length because older adults completed the ACE-III-Mini. Our methods and analysis plan were preregistered (osf.io/m64px).

### Materials and procedure

The study had a mixed design, with age group (younger or older) as a between-subjects factor, and a within-subjects factor of verbalization: silence, overt labeling (labeling colors out loud) and suppression (repeating ‘ba-ba-ba’ out loud). All participants completed one block in each verbalization condition, and each block consisted of 50 trials, resulting in a total of 150 trials per participant. Block order was counterbalanced among participants, such that five participants in each age group performed each of the six possible block order combinations. For the two blocks requiring vocalization, we instructed participants to speak at normal conversational volume. Participants were tested individually. The experimenter remained in the room to ensure that participants followed instructions. All participants performed three practice trials in whichever condition they started with, before starting the experiment. If they did not understand the task, they did further practice.

**Fig. 1:**
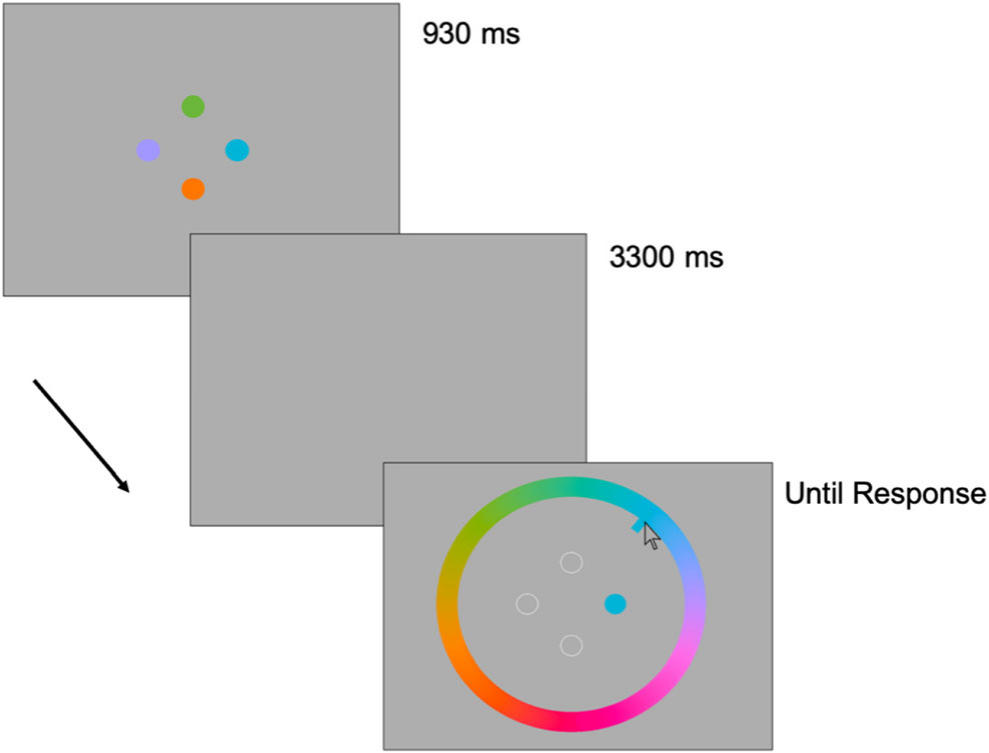
Outline of a typical trial. This is a visual representation where items are not drawn to scale. The function used to generate the color values are available in the Supplementary Materials. (Color figure online)

Each trial started with an instruction text appropriate for the current verbalization block: ‘*Trial Starting’*, ‘*Name Colours’* or *‘Ba-Ba-Ba’*. The participant pressed a key to start each trial. Then, four colored circles appeared simultaneously for 930 ms, on a grey background. This presentation time was longer than the 250 ms used by Souza and Skóra (2017), to ensure that our older adult participants would be able to perceive and label all four colors (see Fig. 1 for an outline of a typical trial). The circles were evenly spaced at 90^o^, 180^o^, 270^o^ and 360^o^ angles around a larger imaginary circle (radius = 150 pixels), and each circle had a radius of 30 pixels (corresponding roughly to Souza & Skóra’s circle radius; 1.6° visual angle, and imaginary circle radius; 6.65° for our screen size).

The colors of the circles presented in the memory task were randomly chosen on each trial, selected from 360 possible color values. The 360 color values were evenly distributed in a circle in the CIELAB color space,^2^ centered in the color space with *L* = 50, *a* = 20, *b* = 20, radius = 60, and then converted to RGB values, trimming nonsense values (see Supplementary Materials). Next, they saw a grey interstimulus interval (ISI) screen of 3,300 ms, followed by the color-wheel and outlines of the four circles. One of the circles was filled in dark grey, probing memory for the first item. Participants responded by clicking the mouse cursor on the shade in the color-wheel they recalled having been in that circle. A second color was then probed, and so on, until participants had recreated all four original colors from memory, probed in a random order. On each trial, the color-wheel rotated randomly (e.g., the pink end of the spectrum might be at the top in one trial, and somewhere else in the next trial). In the suppression condition, participants were instructed to say ‘ba-ba-ba’ while they saw the colored circles and during the ISI, but to respond in silence to ensure articulatory demands during the response phase were similar between conditions (see Souza & Skóra, 2017). During overt labeling, we told participants to label the colors out loud as soon as they appeared on the screen and to use whatever labels they found suitable.

Stimuli were presented using PsychoPy2 (Version 1.82.01; Peirce, 2007) and displayed on a 22-in. LCD monitor, with a diagonal of 20.6-in., and a screen resolution of 1,680 × 1,050. Participants sat at approximately 50 cm unconstrained viewing distance from the screen. After completing the silent block, we asked participants: ‘Did you use a strategy to help you remember the colors? If so, could you please describe that strategy?’ We scored strategies including either ‘naming’, ‘labeling’, ‘saying’, ‘repeating’ or ‘mumbling’ as using a labeling strategy. Strategies which did not include these terms were not scored as labeling.

#### Data analysis

**Fig. 2:**
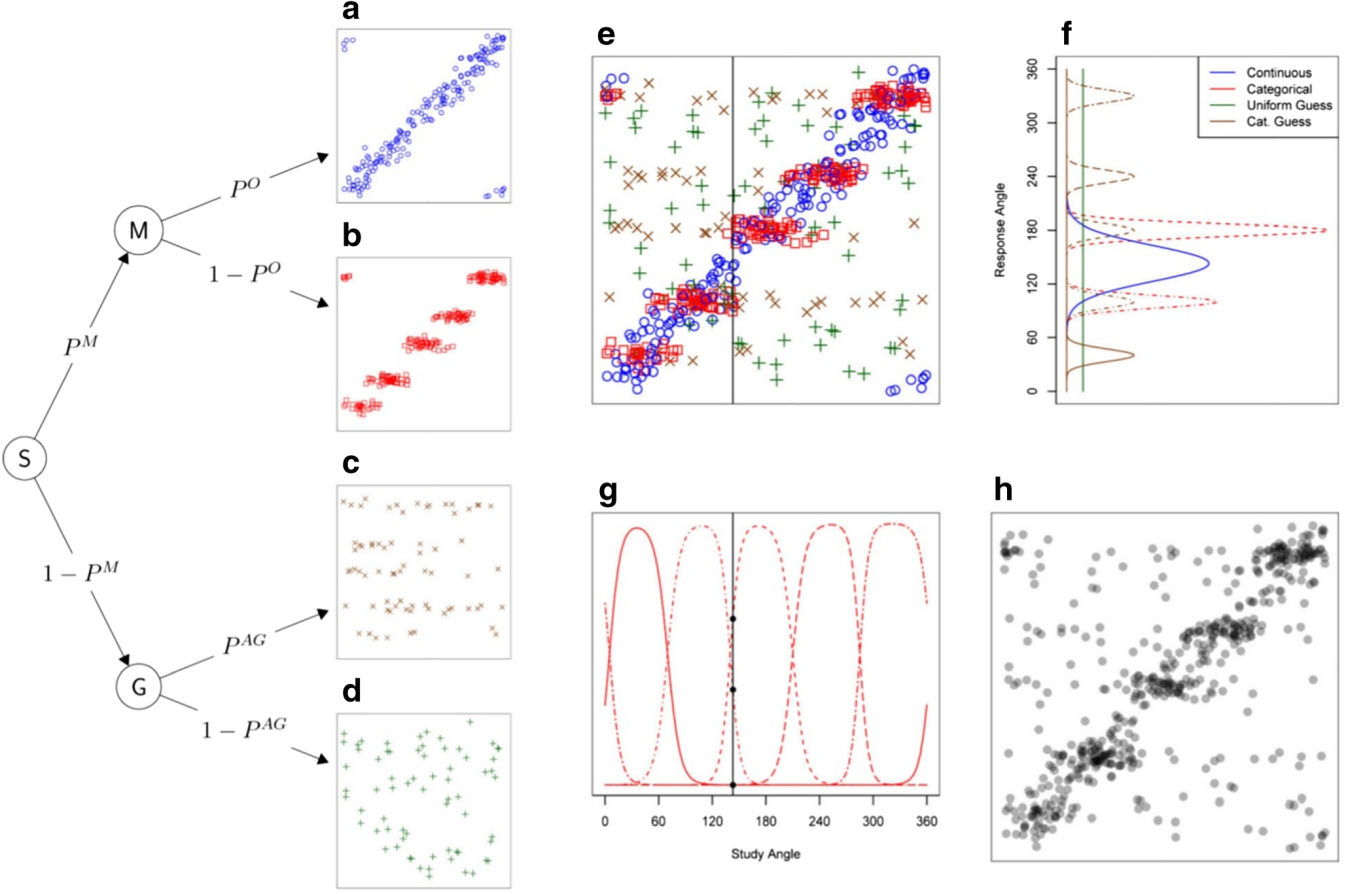
Multinomial process tree for the model and related plots for Hardman et al.’s (2017) categorical-continuous model. For all scatterplots, the *x*-axis represents the studied color hue and the *y*-axis the response hue. **a** Continuous memory: responses vary linearly with the studied hue. The width of the diagonal line indicates continuous imprecision. **b** Categorical memory: for a range of studied hues, the same categorical response is provided. The width of the categorical bands reflects categorical imprecision. **c** Categorical guessing: guessing is distributed over categories. **d** Random guessing. **e** show the points in panels **a–d** combined. **f** Shows response densities for the four different response types for a single study angle (indicated by vertical line in **e**). **g** Shows the function giving the probability that a given study angle will be assigned to the given category. **h** Illustrates the same points as in **e**, but without information about the response type. Reprinted from “Categorical Working Memory Representations Are Used in Delayed Estimation of Continuous Colors,” in K. O. Hardman, E. Vergauwe, and T. J. Ricker, 2017, *Journal of Experimental Psychology: Human Perception and Performance*, *43*(1), 30. (Color figure online)

We used a categorical–continuous mixture model (CatContModel; Hardman et al., 2017) to analyze the data. This model estimates the proportions of colors remembered categorically (participants remember coarse representations, such as ‘red’, that tend to cluster around a few canonical values) versus continuously (participants remember the specific shade of the color) in the delayed-response color-wheel paradigm. Specifically, responses in this task are assumed to be informed either by memory or guessing. If the studied color is a light shade of pink, responses based primarily on categorically labeling it ‘pink’ should cluster around a specific number of canonical values (see Fig. 2b). Alternatively, responses could be informed by continuous, more fine-grained representations of the specific hue, varying linearly with the studied colors (see Fig. 2a). This representation would include information about the particular studied color—for instance, that it was a lighter pink. Storage of continuous information can be more or less fine-grained, and this memory precision is measured by the ‘continuous imprecision’ parameter, represented by the width of the diagonal line in Fig. 2a (equivalent to the imprecision parameter proposed by Zhang & Luck, 2008). In contrast, if responses are not informed by memory, they are classified as guessing. Guesses can be random (continuous guessing, see Fig. 2d), or in accordance with certain categories (categorical guessing, see Fig. 2c). The CatContModel classifies responses into these categories based on probabilistic mixture modeling. This is done by estimating the number of categories and their mean values for each participant using their overall response patterns. Figure 2h shows all responses from an imaginary participant. This imaginary participant has five color categories. The heights of the distributions in Fig. 2f show the likelihood that different response angles would be chosen for a specific study angle, for each of the four response types. Figure 2e illustrates all the responses classified into the four different categories. Figure 2 also shows the multinomial process tree for the model (see the left part of the figure): S represents the start node, and the first branch depends on whether the participant had the tested item in WM, which happens with probability P^M^. If so, they reach node M (memory). If not, they reach node G (guessing). Remembered items can be stored with continuous information—which happens with probability P^O^—corresponding to the response distribution illustrated in Fig. 2a. In contrast, the probability that the memory representation was categorical equals 1 − P^O^. When the item is not remembered, the model assumes that the participant will guess (probability of 1 − P^M^). The response distribution of categorical guessing is illustrated in Fig. 2c, and uniform (continuous) guessing in Fig. 2d. Both guessing distributions are independent of the study angle, while the response distributions are not. Thus, over all responses given by all participants, the model can estimate the following three parameters:

1. The probability that responses were informed by memory (P^M^)
2. The probability that memory information was continuous (P^O^)
3. The precision of the continuous information in memory (σ^O^)

Simply put, P^M^ is the estimated probability of remembering (either categorical or continuous responses) as opposed to guessing. P^O^ is the probability of responding using continuous representations rather than categorical (i.e., informed by precise visual memory representation rather than clustering around a category center), and σ^O^ is the estimated precision of the responses classified as continuous. Here, we test how our experimental manipulations (silence, labeling, or suppression) affected these three parameters in the two age groups. Furthermore, we used these parameters to calculate estimates of WM capacity (K). Regular K is a measure of WM Capacity where capacity (K) represents the total items in memory:

$$Total\ K=\kern0.5em {P}^M\times Set\ Size$$

If capacity is truly four items, P^M^ for four items would equal 1, but when shown five items P^M^ would equal 0.8 (4 = P^M^ × 5). In this study, set size was always four items. Categorical and Continuous K can be calculated by combining P^M^; probability of storage, and P^O^; probability that representation was continuous (rather than categorical; 1 − P^O^). Thus,

$$Continuous\ K={P}^M\times {P}^O\times Set\ Size$$

$$Categorical\ K={P}^M\times \left[1-{P}^O\right]\times Set\ Size.$$

These measures allow us to distinguish whether verbalization manipulations caused *shifts* from one type of representation to the other (i.e. the capacity for one decreased, and the other increased) from a scenario where the manipulation increased one type of representation while the other remained the same.

We implemented the CatCont models in a Bayesian Hierarchical Framework (i.e., a model written in multiple levels that estimates the parameters of the posterior distributions using the Bayesian method). All parameter values were determined through Bayesian Markov chain Monte Carlo (MCMC) sampling techniques. MCMC iteration is a method for obtaining information about posterior distributions in Bayesian inference (van Ravenzwaaij, Cassey, & Brown, 2018), commonly used to compute inferential quantities (see Green, 1995; Han & Carlin, 2001). Hierarchical models reflect an assumption that a participant’s parameter values in a given experimental condition are drawn from a population-level normal distribution. We could thus also obtain population-level parameter estimates for each experimental condition and age group, to assess whether they differed. The (young adult) suppression condition was used as a cornerstone in the modeling. We used Bayesian inference to investigate if there was an effect of age group and verbalization on memory. We based inferences on Bayesian hypothesis testing, which combines prior knowledge about the parameter space (the ‘prior’) with knowledge about the parameter space after seeing the data (the ‘posterior’). Hence, Bayesian inference is not based only on the mean parameter estimate, but also its uncertainty (Kruschke, 2011). Parameter uncertainty is described by the 95% credible interval of the posterior distribution, in addition to the mean parameter value. To compare conditions, we used the Savage–Dickey density ratio, a method of obtaining the Bayes factor by dividing the height of the posterior by the height of the prior at the point of interest (Dickey, 1971; Gamerman & Lopes, 2006; Wagenmakers, Lodewyckx, Kuriyal, & Grasman, 2010). Specifically, this provides a ratio of the likelihood of one hypothesis relative to some other hypothesis (e.g., the alternative hypothesis, cf. the null hypothesis: BF_10_). For more details on how BFs are computed for between-subjects designs, see Hardman (2017).

Bayes factors cannot conclusively be interpreted using threshold cutoff points; therefore, some subjective interpretation is inevitable when describing the result. Typically, BF = 1 is considered ‘no evidence’, BF between 1 and 3 is considered ‘anecdotal’ (Wetzels & Wagenmakers, 2012) or ‘not worth more than a bare mention’ (Jeffreys, 1961), and BF greater than 3 is considered ‘substantial’^3^, between 10 and 30 ‘strong’, 30 and 100 ‘very strong’, and over 100 ‘decisive’ evidence (Jeffreys, 1961; Wetzels & Wagenmakers, 2012). However, these labels are arbitrary (see Morey, 2015), so we applied them tentatively and encourage readers to evaluate the strength of evidence for themselves.

## Results

The following analyses were preregistered via the Open Science Framework (osf.io/m64px). No participant had an average error distance more than 90 degrees on the color-wheel circle—which would indicate chance performance—hence, no one was excluded and replaced for that reason. Verbal labeling strategies in the silence block were not reported to different extents by younger (76%) and older adults (70%); χ^2^(1, *N* = 60) = 0.34, *p* = .56. We also conducted some exploratory analyses to test whether there was a differential effect of suppression and/or overt labeling, compared with performance in silence, between self-reported labelers and nonlabelers. We found no clear evidence that participants who reported using labeling in the silence condition differed from those who did not report having labeled (see Supplementary Materials for further details about these analyses).

### Mixture modeling

#### Model fitting

All our CatCont-models had age group (younger or older) and verbalization condition (silence, overt labeling, or suppression) as factors. As specified in the preregistration, we conducted separate models including either the error distance of (1) only the first-probed memory item or (2) all four items. The first-item analysis is similar to traditional visual WM tasks, only probing one item. In contrast, including all items tested the impact of labels despite interference and decay caused by previous responding. Due to word limit constraints, we focus on the traditional analysis in this paper (as it was of most interest for our hypotheses), while the analyses for all four items are presented in the Supplementary Materials.

We fit all models with three parallel chains of 10,000 iterations each, with a burn-in of 2,000 iterations. Before running the models, we ensured that all Metropolis–Hastings acceptance rates were in the acceptable range (about 0.4 to 0.6; see Hardman, 2017), by adjusting the Metropolis–Hastings tuning values and rerunning the parameter estimation. How colors were assigned to categories (category selectivity, σS) and imprecision with which participants selected categories (σA); accounting for motor noise were fixed across verbalization conditions (Souza & Skóra, 2017). However, we allowed these parameters to vary between the age groups. Similarly, the probability of categorical guessing (P^AG^) was fixed across verbalization conditions (similar to Souza & Skóra, 2017), but allowed to vary between age groups, to allow for the possibility that younger and older adults may rely on such guessing to different extents. Souza and Skóra (2017) collected information about the numbers of categories participants used by recording participants’ labeling out loud. They compared using that maximum category number with letting the model freely estimate the number of categories for each participant and found similar results. Here, we did not record labels,^4^ so we let the model freely estimate the number of categories and their means for each individual (using the default maximum number of 16 categories; Hardman, 2017).

First, we assessed the fits of the two types of CatCont models, the *between-item* model variant (models an individual response as based on *either* a continuous or categorical representation) and the *within-item* model variant (models both kinds of representations as available and combined to produce responses; see also Bae et al., 2015; Donkin et al., 2015). We compared model fit of CatContModel variants using the Watanabe–Akaike information criterion (WAIC), as recommended by Hardman (2017). The between-item model had a smaller WAIC than the within-item model (Δ = −190.7), indicating a better fit to the data. Therefore, we only discuss the results of this model (see Supplementary Materials for output from the within-item models).

### Memory performance: Parameter estimates

**Fig. 3:**
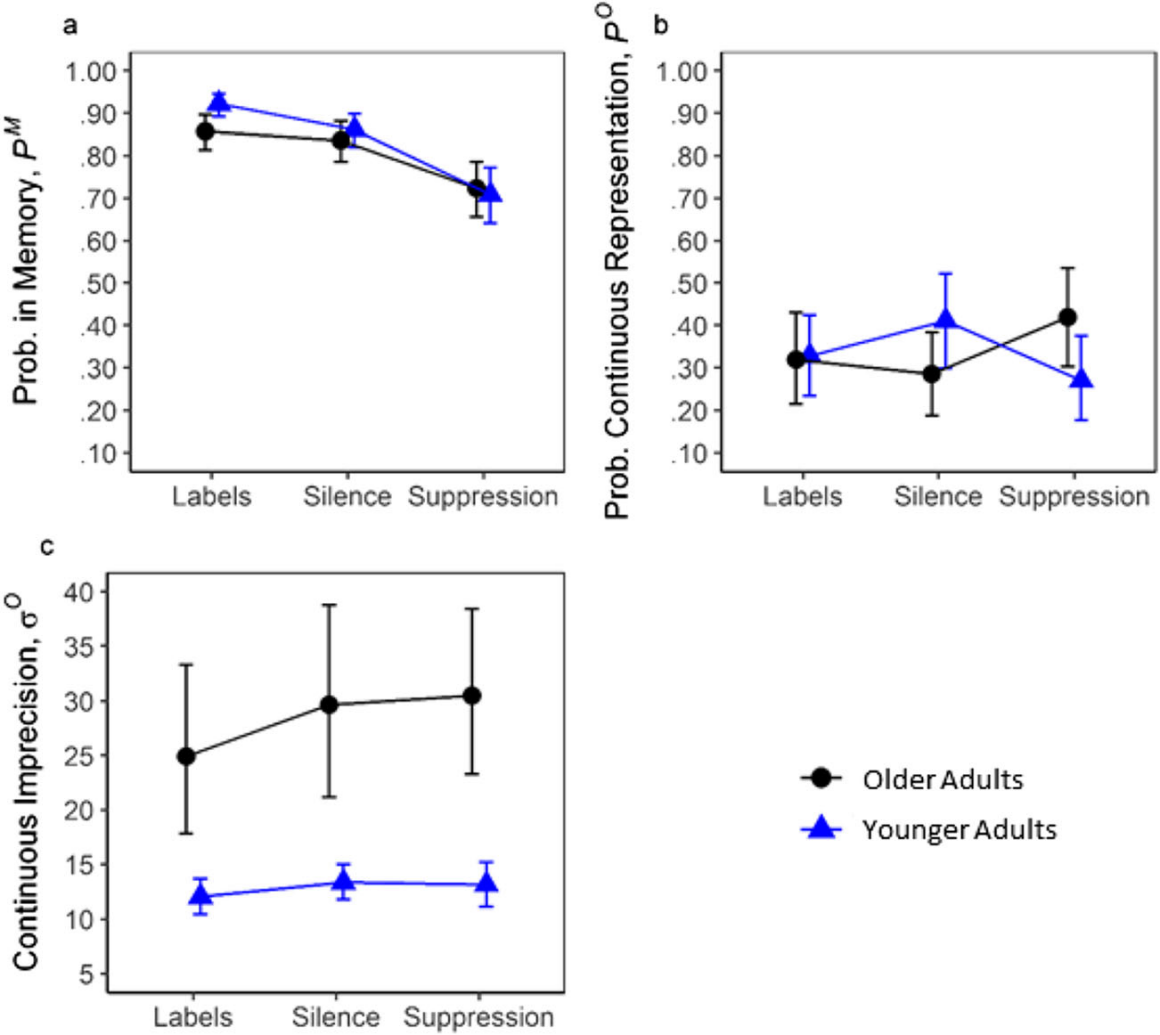
Memory for the first item only. **a** The group-level probability of having the probed item in memory. **b** The group-level probability that memory representation is continuous. **c** The imprecision of the group-level continuous memory representation. Error bars depict 95% credible intervals of the parameters

Figure 3 shows the group-level probability that the first-presented items were in memory (Fig. 3a), the group-level probability that they were stored continuously (Fig. 3b), and the group-level imprecision of continuous representations (Fig. 3c), by age group and verbalization condition. See Table 1 for BF_10_s for the factors and interactions, and Table 2 for BF_10_s for all subset comparisons. There was no evidence here that the probability that the items were remembered (P^M^) differed in the age groups. There was a large effect of verbalization and weak ‘anecdotal’ support for an Age Group × Verbalization interaction. However, older adults did not appear comparatively more impaired under suppression, as suppression impaired the younger adults’ performance comparatively more, when compared with labeling (see Fig. 3; also confirmed by subset analysis contrasting suppression with labeling; Age Group × Verbalization BF_10_ = 29.06; see Table 2). Alternatively, this difference can also be interpreted as younger adults being comparatively more able to benefit from overt labeling, than older adults. Or, both these effects could coexist. There was no main effect of age or verbalization on the probability of continuous (as opposed to categorical) responding. However, there was some evidence for an interaction between age group and verbalization (BF_10_ = 3.50), suggesting that the verbalization instructions affected the proportions of continuous responding differently in the two age groups. There was a ‘decisive’ main effect of age on precision (BF_10_ = 4.35 × 10^3^), such that the older adults’ responses were less precise. The effect of verbalization on precision was ‘anecdotal’, with no evidence for an interaction with age.

**Table 1.** BF_10_s for the effects of the experimental factors

Predictor	Parameter		
	Probability memory (P^M^)	Probability continuous (P^O^)	Continuous imprecision (σ^O^)
*First-presented item only*			
Age group	.16	0.090	4.35 × 10^3^
Verbalization	4.84 × 10^5^	.018	2.58
Age Group × Verbalization	1.53	3.50	.354

**Table 2.** BF_10_s for the effects of subset analyses of the verbalization manipulation

Predictor	Parameter		
	Probability memory (P^M^)	Probability continuous (P^O^)	Continuous imprecision (σ^O^)
First response only			
*Silence vs. suppression*			
Age group	.079	.10	7.90 × 10^3^
Verbalization	9.98 × 10^6^	.13	.38
Age Group × Verbalization	.045	210.36	.15
*Silence vs. labeling*			
Age group	0.39	0.13	407.05
Verbalization	42.03	0.079	3.02
Age Group × Verbalization	.14	0.053	0.20
*Suppression vs. labeling*			
Age group	.208	.18	5.76 × 10^3^
Verbalization	3.11 × 10^11^	.16	5.55
Age Group × Verbalization	29.06	.90	1.85

### WM capacity: Categorical versus Continuous K

**Fig. 4:**
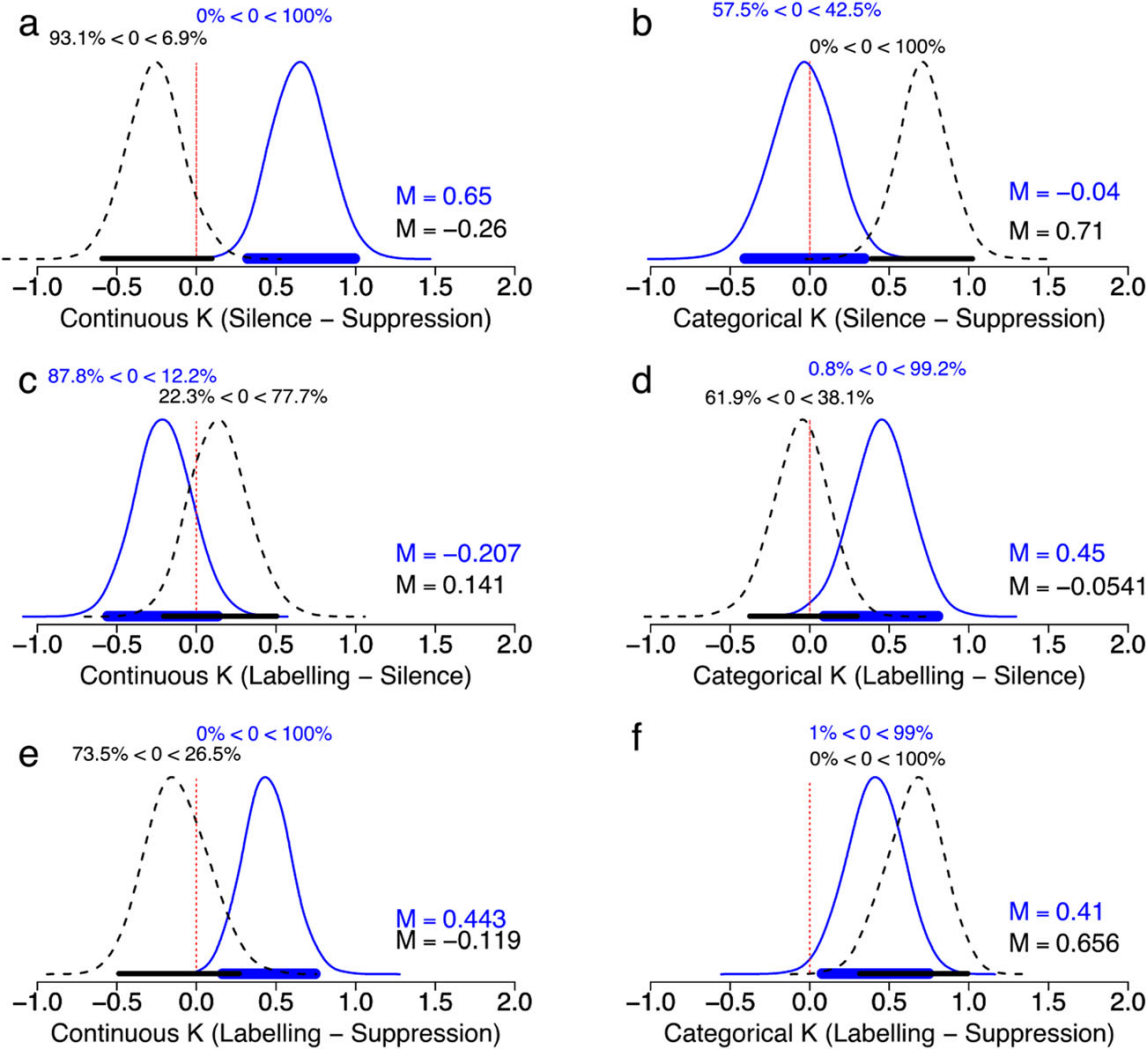
Mean values (M) larger than zero for condition (**a** - **b**, e.g., Silence - Suppression) indicates larger estimates in Condition **a** than **b**).

**Fig. 5:**
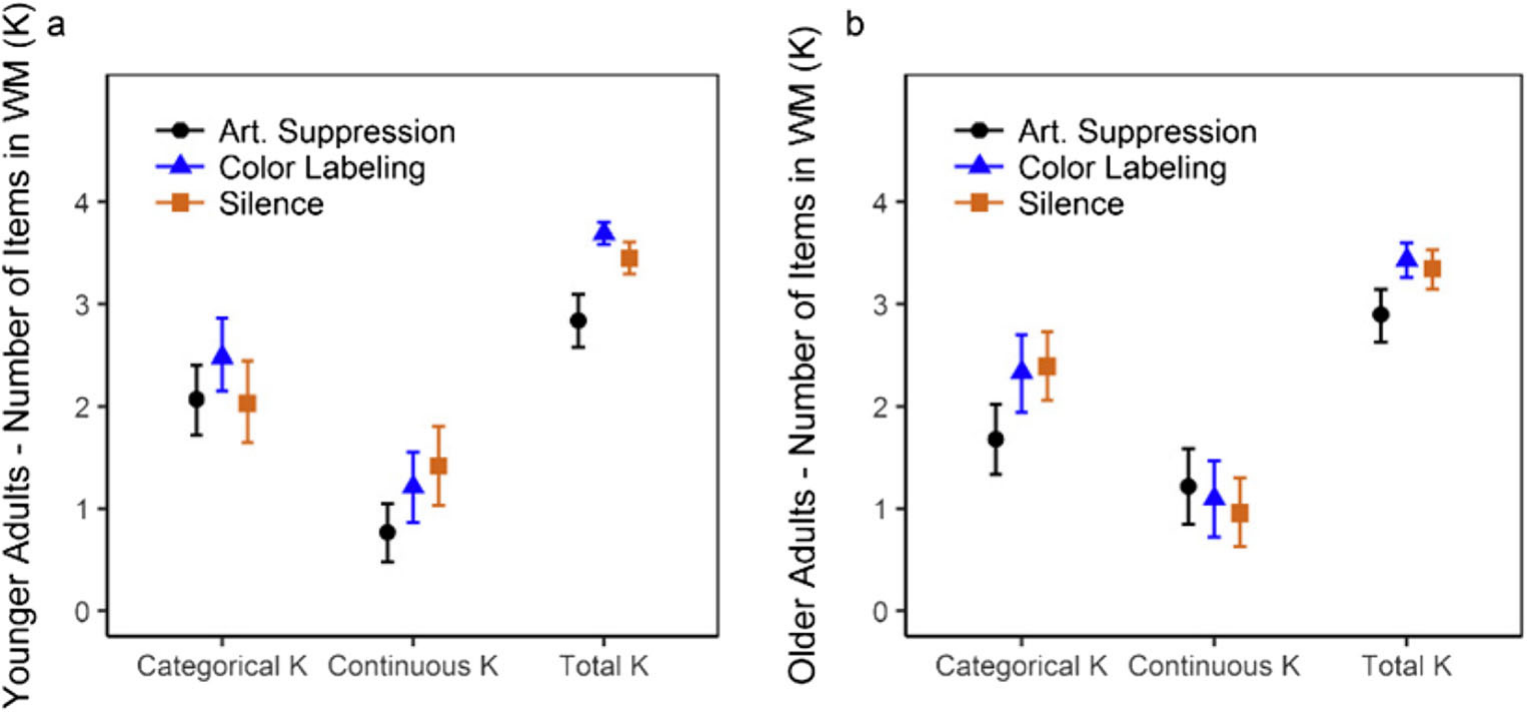
Memory for the first item only. Categorical, continuous, and total K, by age group and verbalization condition. Error bars depict 95% credible intervals

We also calculated estimated Categorical versus Continuous K, for each age group and verbalization condition. Categorical K (*P*^*M*^ × [1 − *P*^*O*^] × *Set Size*) is the estimated capacity for categorical representations, while Continuous K (*P*^*M*^ × *P*^*O*^ × *Set Size*) is the estimated memory capacity for continuous information, in a given condition. With these estimates, we tested whether labeling was associated with greater continuous or categorical capacity—or both—and distinguished among different hypotheses outlined above regarding the labeling benefit in the two age groups: (1) *The categorical visual LTM hypothesis*: labeling increases both Continuous and Categorical Ks, as well as Total K. (2) *The verbal recoding hypothesis:* labeling results in recoding of visual information to a verbal trace, which is used instead of the continuous (‘visual’) representation, resulting ideally in all Categorical K and no Continuous K, with no change in total K. (3) *The dual-trace [visual + verbal] hypothesis*: labeling provides a second, categorical and verbal trace, resulting in increased categorical and Total K, but no change in Continuous K. Posterior differences in Categorical and Continuous K by verbalization condition and age group are presented in Fig. 4. To test differences in labeling in the age groups compared with silence, we compared *prevented labeling* (silence vs. suppression) and *enforced labeling* (silence vs. labeling). Finally—to avoid potential confounds of age-related differences in spontaneous subvocal labeling when performing the task in silence we quantified the labeling benefit by comparing labeling versus suppression (see Fig. 5 for estimates of total, Categorical and Continuous K).

### Preventing silent labeling (silence vs. suppression)

There was a ‘decisive’ main effect of suppression on the probability of remembering (P^M^), such that participants remembered better when doing the task in silence than under suppression (BF_10_ = 9.98 × 10^6^; see Table 2). This was true for both age groups, with no evidence of an interaction. There was no clear main effect of suppression on the probability of having a continuous representation (P^O^), but there was ‘decisive’ evidence for an interaction with age group (BF_10_ = 210.36). Specifically, the proportion of continuous representations under suppression relative to silence decreased for younger adults but increased for older adults (see Fig. 3a). Although this was the case for the proportion of continuous representations, when considering the absolute capacity, Continuous K did not change credibly under suppression in older adults (*M* = +0.26 items). In younger adults, however, it was credibly reduced under suppression (*M* = −0.65; see Fig. 4a). Categorical memory representations in older adults were credibly reduced by suppression (*M* = −0.71 items), but not in younger adults (*M* = −0.04 items; see Fig. 4b). Suppression did not have a conclusive effect on precision (BF_10_ = .38) and did not appear to affect precision differently in younger and older adults (BF_10_ = .15).

### Enforcing labeling (silence vs. labeling)

Overt labeling improved memory (P^M^) compared with performing the task in silence (BF_10_ = 42.03), in both age groups (there was no evidence for an interaction with age; BF_10_ = .14). It did not influence the probability of having a continuous (as opposed to categorical) representation of the first item (P^O^), regardless of age group (see Table 2). Labeling did not produce a credible change in Continuous K in either age group (see Fig. 4c). However, compared with performance in silence, younger adults’ categorical memory representations increased credibly when instructed to label (*M* = +0.45 items; see Fig. 4d). In contrast, older adults’ categorical memory capacity under instructed labeling did not differ credibly from their performance in silence (see Fig. 4d). Furthermore, there was some evidence that overt labeling increased precision (i.e., reduced imprecision; σ^O^) for the first item (BF_10_ = 3.02), but not to different extents in the age groups.

### The labeling benefit (labeling vs. suppression)

Overt labeling improved memory (P^M^) in both age groups compared with suppression (BF_10_ = 3.11 × 10^11^). There was no evidence of any effect of labeling on the probability of continuous (as opposed to categorical) responding (P^O^). However, the absolute benefit associated with labeling was due to a boost in Categorical K in both age groups (younger *M* = +0.41, older *M* = +0.65 items; see Fig. 4f). However, while this was the only source of the boost in older adults, the younger adults’ Continuous K also increased credibly (*M* = +0.44 items; see Fig. 4e). Thus, similar to Souza and Skóra (2017), our younger adults’ labeling benefit fit best with the categorical long-term memory hypothesis. In contrast, the older adults’ Continuous K increased slightly under suppression (*M* = +0.12 items). However, this increase was not within the 95% credible interval (see Fig. 4e), which would be required to support the verbal recoding hypothesis (i.e., the number of verbal representations increases at the expense of the visual representation). Instead, the older adults’ gain fit best with the dual-trace hypothesis; labeling is beneficial because it adds a verbal (categorical) representation to the visual WM trace, without changing the number of visual representations*.* Furthermore, there was some evidence that suppression reduced precision (i.e., increased imprecision σ^O^) for the first item (BF_10_ = 5.55), but unclear whether this occurred to different extents in the age groups (Age Group × Verbalization; BF_10_ = 1.85).

### Consistency check

Finally, we analyzed data from only those participants who completed the silence block before being introduced to the overt labeling manipulation (*N* = 32), to test whether results were driven by participants changing how they performed the task in silence after exposure to instructed labeling. Results generally appeared similar, apart from no evidence for age difference in precision, and less evidence for an Age Group × Verbalization interaction for suppression versus silence comparison for continuous responding (P^O^). However, Categorical versus Continuous K comparisons were similar to the original results (see Supplementary Materials), suggesting that being exposed to the labeling instruction may have increased age group differences, but differences were still present in participants who were unaware of the labeling instruction.

## Discussion

Following evidence that verbal labeling improved visual WM performance by boosting the number of categorical and continuous representations, as well as precision of continuous representations in young adults (Souza & Skóra, 2017), we investigated if labeling would have a similar effect in healthy older adults. Evidence of comparatively less impaired verbal WM in older adults (e.g., Jenkins et al., 2000; Park et al., 2002) and suggestions that participants of different age groups may rely on different cognitive capacities to perform the same task (Johnson et al., 2010; Reuter-Lorenz, 2002) made us question the ‘dull hypothesis’ that older adults perform just like younger adults, but more poorly. We addressed the following questions: Do older adults (1) Spontaneously use verbal labels more than younger adults when performing the task in silence? (2) Depend more on verbal labels for visual memory performance than younger adults? (3) Benefit from verbal labels in the same way as younger adults?

### Spontaneous use of verbal labels

We tested if younger and older adults differed in the extent to which they spontaneously applied subvocal labeling to this visual WM task using the following logic: If participants used verbal labels in silence, performance during silence and labeling should be similar, but different under suppression (when labels cannot be used). Hence, if the proportion of categorical and continuous representations differs from that in silence under either verbalization instruction (labeling or suppression), it suggests that the manipulated condition differed from spontaneous performance.

Evidence that manipulating subvocal labeling (silence vs. suppression) affected the age groups’ probabilities to respond continuously differently was ‘decisive’. Specifically, compared with when performing the task in silence, younger adults’ categorical memory representations increased credibly when instructed to label, but were not reduced by suppression—suggesting that they were not consistently labeling subvocally in silence. In contrast, older adults’ categorical memory capacity in silence did not differ credibly from their performance under instructed labeling (see Fig. 4d) but was poorer under suppression (see Fig. 4b)—consistent with spontaneous subvocal labeling in silence. In sum, the older adults lost categorical representations under suppression, while the younger adults gained categorical representations when instructed to label, compared with silence. Combined, these observations suggested that older adults spontaneously (i.e., in the silence condition) used verbal labels to maintain coarse, categorical representations more than the younger adults, and furthermore that these representations were maintained via subvocal labeling, since suppression reduced them.

These different tendencies to rely on different types of representations in silence were not detected when comparing the overall memory performance between age groups (P^M^; which combines both continuous and categorical representations; Table 1). Moreover, similar proportions of younger and older adults reported using verbal strategies in the silence condition. Hence, these age differences likely go unnoticed in visual WM tasks.

Previous research has found that younger participants can control the trade-off between quality and quantity in some visual WM tasks via verbal encoding (e.g., Ramaty & Luria, 2018; Zhang & Luck, 2011). If reliance on such verbal encoding differs systematically between age-groups—as our results indicate—this could be problematic for age-comparisons in a variety of visual WM tasks, for instance paradigms measuring visual feature-binding, if remembering individual features lends itself to such labeling and remembering bindings does not (Brockmole & Logie, 2013).

More broadly, endeavors to measure neural states that correspond to mental codes are central to hypotheses in cognitive psychology (Haxby et al., 2001; Haynes & Rees, 2006; Lewis-Peacock & Postle, 2012; Lewis-Peacock et al., 2014). However, tasks based on the same visual stimuli have been observed to elicit different activity depending on which strategy participants were instructed to use (Decety et al., 1997). Older adults appear to activate less, more, or even different neural structures than younger adults when performing a memory task (see Cabeza, 2002; Park, Polk, Mikels, Taylor, & Marshuetz, 2001), thought to reflect compensatory recruitment (Cabeza, 2002; Cherry, Park, & Donaldson, 1993; Park et al., 2001). Our results highlighted the importance of establishing the extent to which differences are driven by age-related strategic preferences in approaches to visual memory tasks, and that such differences may be detected using mixture modeling combined with explicit labeling manipulations.

### Do older adults depend more on verbal labels for visual memory?

We tested whether older adults’ memory (P^M^) would be comparatively more impaired during concurrent suppression compared with the two other conditions (i.e., when labeling was prevented). This was not supported. Instead, we observed strong evidence that suppression impaired the younger adults’ performance comparatively more when contrasting it with labeling. This Age × Labeling interaction left it unclear whether younger adults were able to benefit more from labeling, or were comparatively more disrupted by suppression. Either way, these results contradicted the idea that older adults depend on a less impaired verbal store to compensate for reduced visual memory capacity, since, if so, their overall performance should have deteriorated more than that of the younger adults under suppression. Thus, interestingly, older adults’ increased tendency to use verbal labels when performing the task silently (as discussed above) did not appear to correspond to a *need* to use such labels to maintain good overall memory performance, compared with younger adults.

Moreover, older adults’ continuous representations were less precise than the younger adults’. Our results indicated that age-related decline in precision was not simply due to greater reliance on categorical (‘verbal’) representations in older adults, since we observed a substantial age-related decline in the precision of continuous representations even when categorical representations were separated out by the CatContModel (i.e., the imprecision parameter analyzed in this paper should not be influenced by categorical responding)—supporting the notion that declining visual WM precision is an important feature of cognitive aging (Peich et al., 2013). However, we did not find strong evidence for an age effect on precision in the consistency check analysis, which only included participants who had not been exposed to the overt labeling condition prior to the silence block. Bayes factors close to 1 may suggest insufficient data in this reduced sample size (see Dienes, 2014).

### Do older and younger adults benefit from verbal labels in the same way?

To measure memory gain associated with labeling in the two age groups without potential confounds of personal or age-related preferences, we compared instructed overt labeling with suppression (prevented labeling) and found that older adults appeared to benefit differently from verbal labels. We compared the influence of labeling on three aspects of memory performance: categorical representations, continuous representations, and increased precision of continuous representations. Souza and Skóra (2017) observed that verbal labels improved all three in young adults. They concluded that labels boosted memory by activating categorical visual LTM, rather than simply improving memory by providing extra, verbal traces. Several other studies have also suggested that Categorical LTM (i.e., preexisting visual representations; Brady, Konkle, & Alvarez, 2011) can boost visual (continuous) WM (Alvarez & Cavanagh, 2004; Buttle & Raymond, 2003; Curby & Gauthier, 2007; Curby, Glazek, & Gauthierm, 2009; Olsson & Poom, 2005; Sørensen & Kyllingsbæk, 2012; but see also Pashler, 1988). For younger adults, we replicated these observations. In contrast, older adults did not appear to gain continuous representations when labeling. Instead, the additional information associated with labeling was primarily categorical, suggesting that older adults’ gains associated with labeling were verbal in nature (consistent with the dual-trace [visual + verbal] hypothesis).

However, relative to silence as a baseline, we found that overt labeling improved categorical capacity while suppression reduced continuous capacity in younger adults (see Fig. 5)*.* This highlighted a potential alternative explanation behind the labeling benefit in younger adults. Instead of verbal labels activating categorical visual LTM (Souza & Skóra, 2017), the greater continuous contribution to performance associated with labeling could be because suppression reduced visual memory capacity during encoding, perhaps by draining a general resource (Cowan, 2005; Ma et al., 2014). If so, this effect may not be noticeable in studies using categorical stimuli. It is also likely that our experimental conditions reflect different degrees of labeling (overt labeling: nearly always, silence: sometimes, suppression: less often). If the effect of labeling on Categorical and Continuous K differs in magnitude, and silence is not a true middle ground (which is unlikely), this might also explain this pattern of results. This highlights the difficulty associated with attempting to prevent labeling such that processing demands are equal between conditions, and is a limitation of this paradigm. However, referring to performance in silence when investigating the labeling benefit is problematic. Instructed labeling might disrupt other processes occurring in unrestrained conditions. For example, perceptual grouping based on what people might label as ‘warm’ or ‘cool’ colors might be one such process, which has been found to influence memory for individual items even in randomly selected to-be-remembered arrays of colors (Alvarez, 2011; Brady & Alvarez, 2011). Overt labeling disrupting some other process would explain why labeling did not improve continuous memory compared with silence in our younger adults. Either way, it appeared that labeling—despite being very beneficial for overall memory in both age groups—affected the types of representations held in memory differently.

### Limitations

All parameter estimates presented in this paper depend on the assumptions of the CatContModel (Hardman, 2017). It is possible that other processes which contribute to responses in the delayed estimation task (e.g., perceptual grouping processes) may not be adequately captured by model. This is a limitation of this research. However, the CatContModel appears to more adequately fit data generated by participants than models which assume that all responses are continuous (see Hardman et al., 2017), and it enabled us to compare categorical versus continuous representations in a way that would not be possible using a set of fixed, categorical to-be-remembered items. Moreover, including a perceptual matching task to ensure that younger and older adults did not differ in memory precision due to for instance greater motor noise in older adults may have been informative. However, Loaiza and Souza (2018) found that while there was an age difference in perceptual matching, it was much smaller than that observed in the WM task, suggesting that it did not fully account for the age differences in WM precision memory (see also, Souza, 2016; Loaiza & Souza, 2019). Also, age-related perceptual and/or motor differences are unlikely to vary between our experimental conditions. Next, there were instances where participants coughed, sneezed, or otherwise lapsed attention during a trial, and may not have said all four colors on that specific trial (or, indeed, maintained suppression perfectly). This would have been a very small minority of trials. However, recording sounds would have allowed us to exclude such trials, and ensure that the number of such instances did not vary between participants in the two age groups. Finally, while the experimenter monitored that color labels were said aloud by all participants in the labeling condition, we did not monitor which label was applied to which color. Thus, it is possible that participants may have used unrelated, random color names simply to comply with the instruction. However, the large beneficial effect of labeling, in both age groups, indicates that this was not the norm, or that assigning any kind of label can be beneficial.

## General discussion

A range of studies have shown that people can retain many mental codes in parallel (the ‘multiple encoding’ hypothesis; e.g., Lewis-Peacock et al., 2014; Logie et al., 2016; Paivio, 1971; Wickens, 1973), and many cognitive theories explicitly model the multidimensional nature of memory representations. Researchers debate how flexibly resources can be shared across the visual and verbal/auditory modalities in memory tasks, as well as whether these modalities are actually distinct in memory: Observations of clear capacity costs due to cross-modal sharing of resources (Morey & Cowan, 2004, 2005; Morey, Cowan, Morey, & Rouder, 2011; Saults & Cowan, 2007; Vergauwe, Barrouillet, & Camos, 2010) conflict with studies in which such costs were not found (e. g., Cocchini et al., 2002; Fougnie & Marois, 2011).

Introduction of delayed estimation tasks enabled fidelity measures of visual WM representations and started intense debates about whether visual WM is best characterized as an information-limited system (Alvarez & Cavanagh, 2004; Wilken & Ma, 2004), or as limited by a predetermined, fixed-item limit (Luck & Vogel, 1997; Zhang & Luck, 2008). WM capacity has usefully been conceptualized as both the number of items that can be stored and the precision with which those items are stored—analogous to storing images on a USB-drive: You can store more images with low resolution or fewer images with very high resolution, given its finite volume (Brady et al., 2011). Our research adds to the body of research highlighting that verbal representations, either as ‘audio files’ or simply as ‘file names’ categorizing visual representations, also need to be acknowledged, and that they may be connected to visual items in complex ways. Participants may use one or other of these forms of representation according to their preferences and ability to use each of them (e.g., Logie, 2018). Indeed, preferences for the type of mental codes (or ‘file formats’) appeared to vary with age. In younger adults, verbal labels may be better conceptualized as file names, useful to open specific files from the hard drive (LTM knowledge), thus activating representations (e.g., how the color red looks), which can support this limited visual storage system (e.g., Olsson & Poom, 2005). In older adults, the benefit of verbal information appeared to act more like an audio file (i.e., maintaining a coarse representation, but not necessarily supporting activation of a stored visual representation). Our results highlighted challenges associated with attempting to study the visual system in isolation (e.g., separate from verbal labels) when comparing younger and older adults, and suggest that these challenges might be addressed by explicitly comparing instructed labeling with suppression, and modeling categorical and continuous responses.

### Conclusion

At first glance, our results appeared consistent with the dull hypothesis (Perfect & Maylor, 2000)—that older adults performed the memory task like less precise younger adults. Indeed, while we found no strong effect of age on the overall probability of remembering (continuous and categorical representations taken together), there were differences in precision, emphasizing its usefulness as a more fine-grained measure of the effects of aging. Older adults’ overall memory was not more impaired when suppression prevented verbal labeling, which suggested that older adults’ visual memory performance did not *depend* more on verbal labeling.

However, older adults appeared to rely more on coarse, categorical representations than younger adults when doing the task in silence. Furthermore, these representations appeared to be supported by subvocal labeling, since they were specifically reduced under suppression. Finally, while we replicated Souza and Skóra’s (2017) finding that labeling benefitted younger adults via activated visual categorical LTM, older adults did not appear to benefit in the same way.

People likely use different kinds of mental codes (e.g., visual and verbal) interchangeably to retain information in memory to navigate day-to-day situations. These results suggested that there are age differences which are not apparent when looking only at overall memory performance, which may be important in understanding age differences in more nuanced visual WM phenomena, such as feature-binding or brain activity, in future research.

## Electronic supplementary material

ESM 1 (DOCX 23807 kb)

## Footnotes

1 We based this sample size on Experiment 3 in Souza and Skóra (2017), since measures of effect sizes are less straightforward to obtain from Bayesian analysis (see Bayarri & Berger, 2004). This experiment was most similar to our design because each participant performed three different conditions, with a set size of four items, with 50 trials in each. In 30 younger adults, they could detect a credible difference between these conditions (e.g., between suppression and overt labeling).

2 The CIELAB color space is a system for the organization of colors, which expresses color as three numerical values, *L** for the lightness and *a** and *b** for the green–red and blue–yellow color components.

3 The common meaning of “substantial” has changed over time (R. D. Morey, 2015).

4 While the experimenter did not monitor or record which specific labels were used, the occurrence of labeling was monitored throughout the session through constant experimenter presence. All participants were able to say four color names in the experimental time frame, as established during the practice session.
