# Supplementary material for: Cognitive aging and verbal labeling in continuous visual memory
Source: Mem Cognit. 2020 May 29;48(7):1196–213. doi: 10.3758/s13421-020-01043-3 (PMC7498490; doi:10.3758/s13421-020-01043-3)
Supplement: Supplementary file 1 — (DOCX 23807 kb) [file 13421_2020_1043_MOESM1_ESM.docx]

**Supplementary Materials**

for *Cognitive Aging and Verbal Labeling in Continuous Visual Memory*

Table S1

*BF_10_s for the effects of the experimental factors for all four responses (between-item model).*

| **Predictor** | **Parameter** | | |
| --- | --- | --- | --- |
|  | **Probability memory (P^M^)** | **Probability continuous (P^O^)** | **Continuous imprecision (σ^O^)** |
|  | | | |
| *All responses* | | | |
| Age Group | 1.81 | .106 | 10.98 |
| Verbalization | 6.14 × 10^18^ | 9.34 | 3.25 × 10^5^ |
| Age Group × Verbalization | 7.28 | 5.05 | .10064 |

Table S2.

*BF_10_s for the effects of subset analyses of the verbalization manipulation, for all four responses (between-item model).*

| **Predictor** | **Parameter** | | | | | |
| --- | --- | --- | --- | --- | --- | --- |
|  | **Probability memory (P^M^)** | | **Probability continuous (P^O^)** | | | **Continuous imprecision (σ^O^)** |
| *Silence vs. Suppression* | |  | |  |  | |
| Age Group  Verbalization  Age Group × Verbalization | | .45  3.19 × 10^21^  5.61 | | .37  60.49  .025 | 5.46  6.06 × 10^4^  .24 | |
| *Silence vs. Labelling*  Age Group  Verbalization  Age Group × Verbalization | | 8.35  1.19 × 10^11^  .013 | | .084  .042  17.02 | 7.04  1.33  .021 | |
| *Suppression vs. Labelling*  Age Group  Verbalization  Age Group × Verbalization | | 1.36  3.01 × 10^38^  118.18 | | .089  350.58  .55 | 13.20  4.68 × 10^8^  .26 | |

Table S3

*BF_10_s for the effects of the experimental factors in the within item models.*

| **Predictor** | **Parameter** | | |
| --- | --- | --- | --- |
|  | **Probability memory (P^M^)** | **Probability continuous (P^O^)** | **Continuous imprecision (σ^O^)** |
| *First response only* | | | |
| Age Group | .28 | .90 | .21 |
| Verbalization | 1.05 × 10^6^ | .27 | 2.59 |
| Age Group × Verbalization | .22 | 2.83 | 4.97 |
|  | | | |
| *All responses* | | | |
| Age Group | 5.35 | .29 | 1.77 |
| Verbalization | 3.49 × 10^20^ | .84 | 4.64 |
| Age Group × Verbalization | 5.65 | 5.61 × 10^6^ | 2.49 |

Table S4.

*BF_10_s for the effects of the experimental factors in the consistency check analysis (excluding participants who did the labeling condition prior to the silence condition).*

| **Predictor** | **Parameter** | | |
| --- | --- | --- | --- |
|  | **Probability memory (P^M^)** | **Probability continuous (P^O^)** | **Continuous imprecision (σ^O^)** |
| *First response only* | | | |
| Age Group | .094 | .15 | .69 |
| Verbalization | 4.20 × 10^5^ | .033 | .95 |
| Age Group × Verbalization | .64 | .38 | .62 |
|  | | | |
| *All responses* | | | |
| Age Group | .13 | .104 | 2.50 |
| Verbalization | 2.40 × 10^16^ | .40 | 1.90 × 10^3^ |
| Age Group × Verbalization | 10.07 | 77.15 | .176 |

Table S5.

*BF_10_s for the effects of subset analyses of the verbalization manipulation for the consistency check analysis (excluding participants who did the labeling condition prior to the silence condition).*

| **Predictor** | **Parameter** | | | | | |
| --- | --- | --- | --- | --- | --- | --- |
|  | **Probability memory (P^M^)** | | **Probability continuous (P^O^)** | | | **Continuous imprecision (σ^O^)** |
| **First response only** | | | | | | |
| *Silence vs. Suppression* | |  | |  |  | |
| Age Group  Verbalization  Age Group × Verbalization | | .13  2.61 × 10^7^  .014 | | .18  .21  2.02 | .98  .44  .22 | |
| *Silence vs. Labeling*  Age Group  Verbalization  Age Group × Verbalization | | .090  1.58 × 10^2^  1.20 | | .11  .11  .044 | .64  .70  .83 | |
| *Suppression vs. Labeling*  Age Group  Verbalization  Age Group × Verbalization | | .12  1.24 × 10^11^  5.62 | | .27  .18  .13 | .66  2.48  1.18 | |

Table S6.

*Posterior means and credible intervals of the following parameters: Number of Active Categories, Categorical Guessing, Categorical Selectivity, Categorical Imprecision for the first-item only, and all-item analyses. These parameters were allowed to vary between age groups, but not between verbalization conditions.* ****


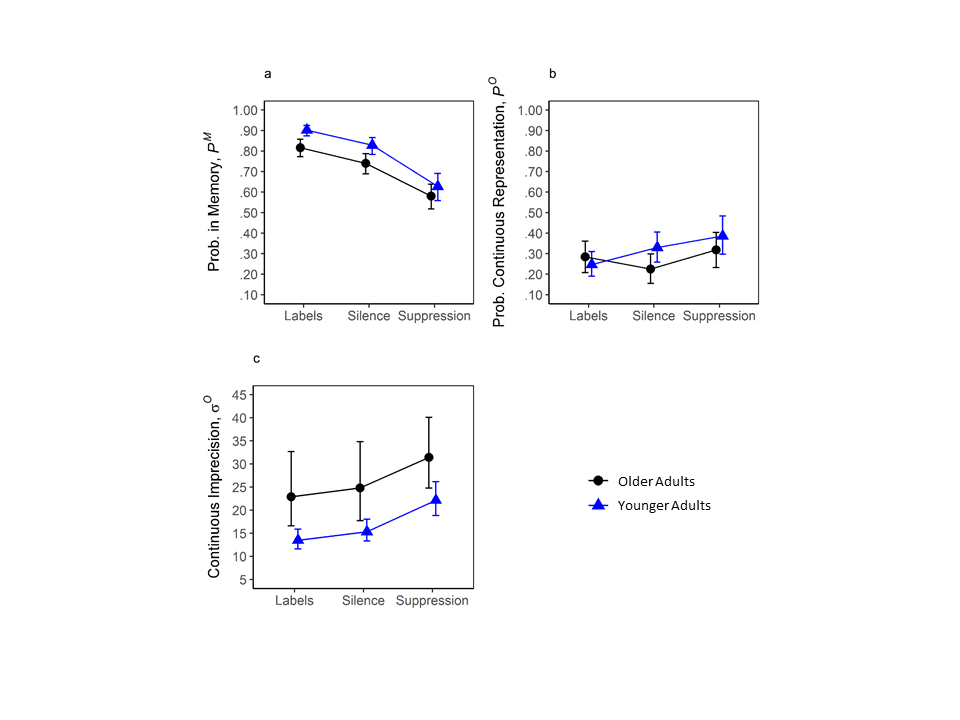


Fig. S1. Memory for all items (between-item model). Panel a. Group-level probability of having the probed item in memory. Panel b. The group-level probability that memory representation is continuous. Panel c. The imprecision of the group-level continuous memory representation.

Fig. S2. Memory for all items (between-item model). Posterior differences in continuous and categorical K for specified comparisons. Mean values (M) larger than 0 for condition (A – B) indicates larger estimates in condition A than B. Each panel presents the percentages of the curves that are above and below 0 (null effect), the means (M), and the 95% credible intervals of the means (bars underneath each curve). Older adults in dotted black, younger in solid blue.


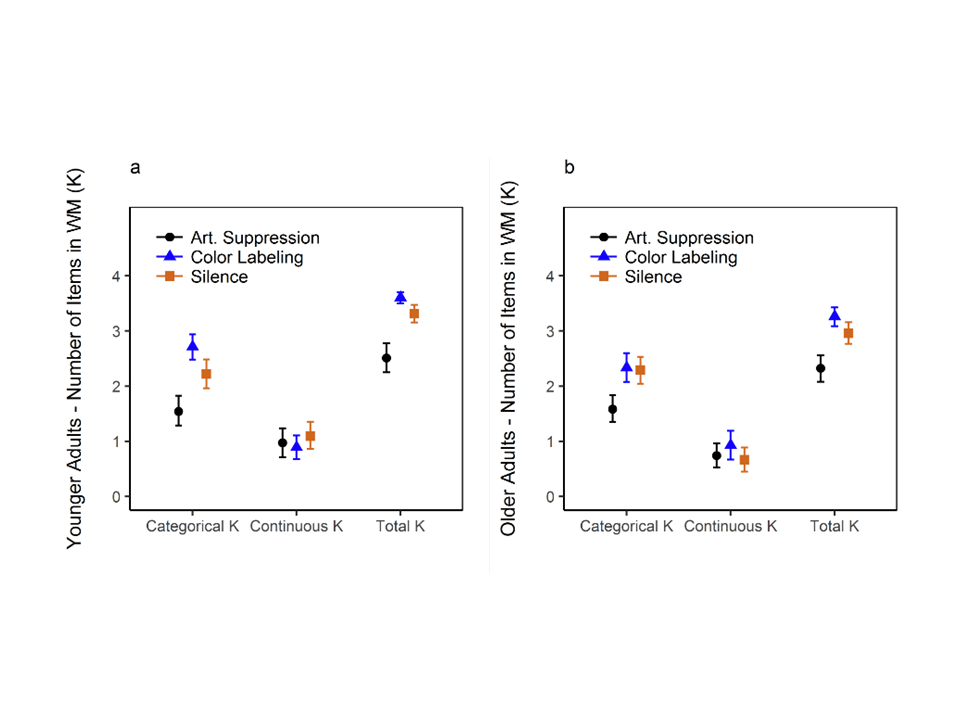
Fig. S3. Memory for all items (between-item model). Categorical, Continuous and Total K, by age group and verbalization condition.

Fig. S4. Memory for the first item only (between-item model), in the consistency check analysis (excluding participants who did the labeling condition prior to the silence condition). Posterior differences in continuous and categorical K for specified comparisons. Mean values (M) larger than 0 for condition (A – B) indicates larger estimates in condition A than B. Each panel presents the percentages of the curves that are above and below 0 (null effect), the means (M), and the 95% credible intervals of the means (bars underneath each curve). Older adults in dotted black, younger in solid blue.

**
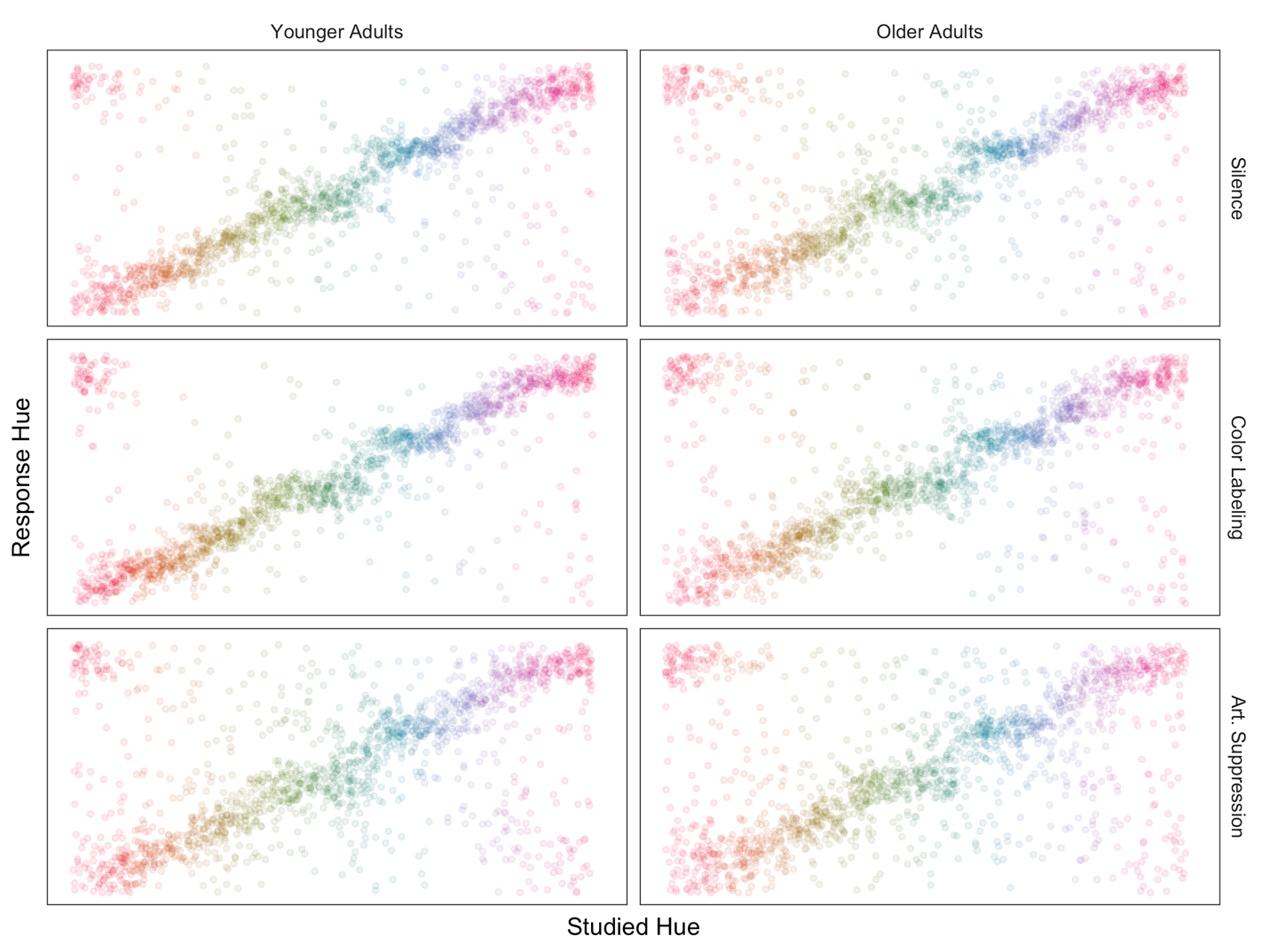
**

Fig. S5. Scatterplot of responses from participants in the two age groups, across the three verbalization conditions, including memory for the first-item only.

**
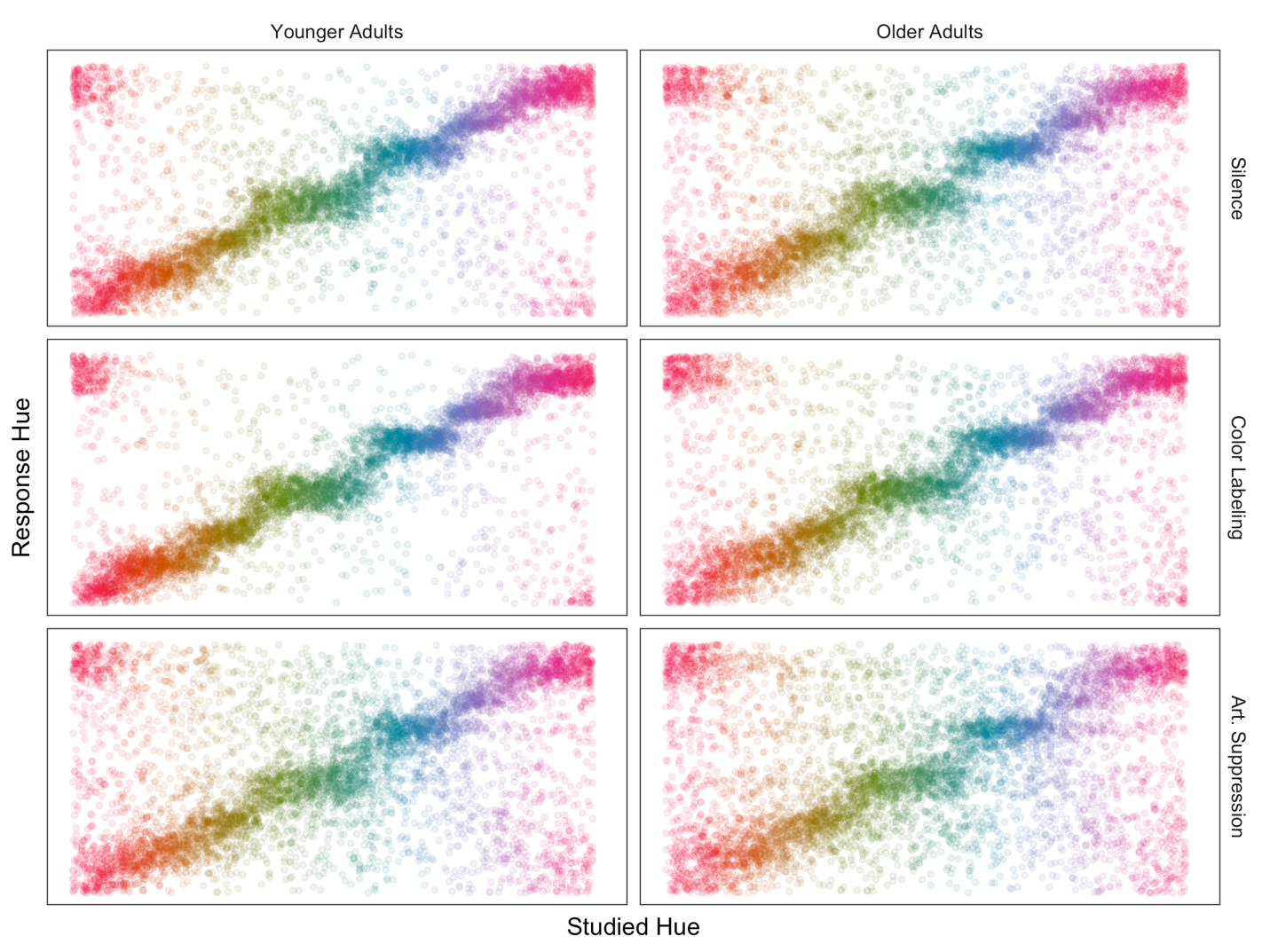
**

Fig. S6. Scatterplot of responses from participants in the two age groups, across the three verbalization conditions, including memory for all four items.


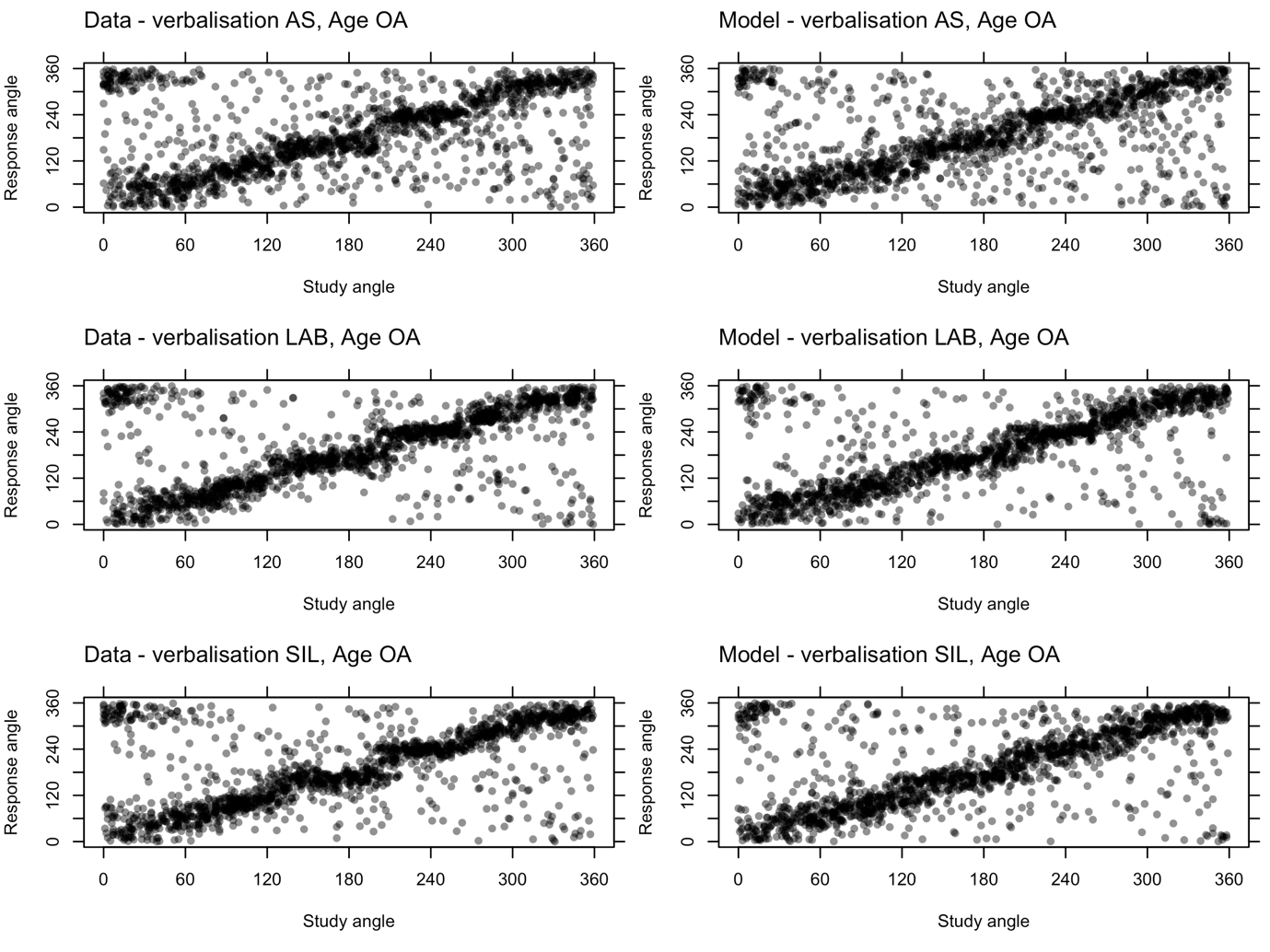


Fig. S7. Posterior Predictive check for older adults in the three verbalization conditions.


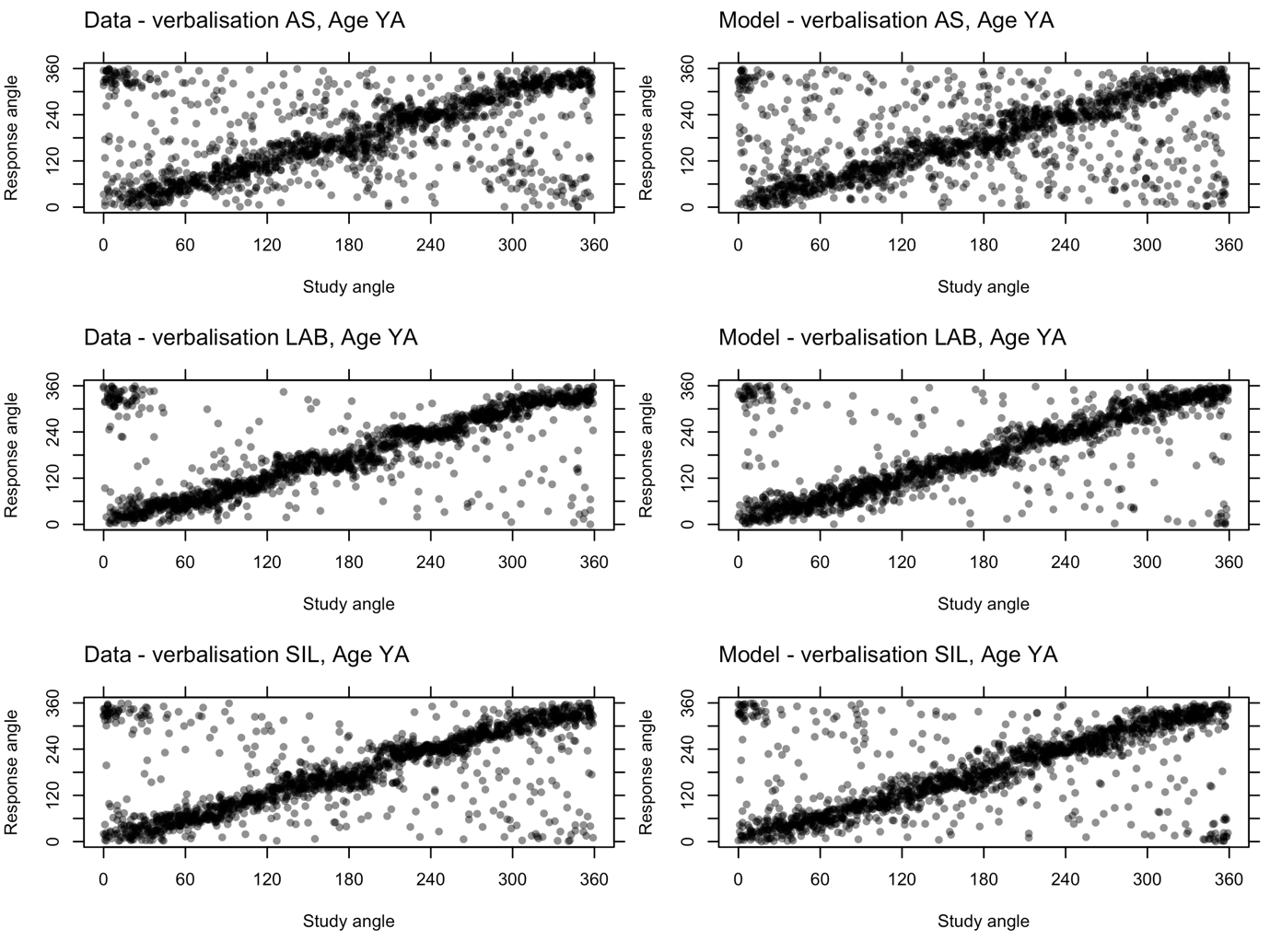


Fig. S8. Posterior Predictive check for younger adults in the three verbalization conditions.

**Function used to generate color values**

def LAB2RGB(L, a, b, radius): # draws a circle in CIELab colour space with specified centre (L, a, b) and raduis then converts to RGB, trimming nonsense values

colours = []

# create CIELab colours

for ang in range(1, 361):

theta = ang * pi / 180.000 # converts angle to radian

A = a + radius*numpy.cos(theta)

B = b + radius*numpy.sin(theta)

# Lab to XYZ

var_Y = (L + 16) / 115.000

var_X = A / 500.000 + var_Y

var_Z = var_Y - B / 200.000

# filter X, Y, Z with threshold 0.008856

if var_Y**3 > 0.008856: var_Y = var_Y**3

else: var_Y = ( var_Y - 16 / 116.000 ) / 7.787

if var_X**3 > 0.008856: var_X = var_X**3

else: var_X = ( var_X - 16 / 116.000 ) / 7.787

if var_Z**3 > 0.008856: var_Z = var_Z**3

else: var_Z = ( var_Z - 16 / 116.000 ) / 7.787

# reference points

ref_X = 95.047

ref_Y = 100.000

ref_Z = 108.883

X = ref_X * var_X / 100.000

Y = ref_Y * var_Y / 100.000

Z = ref_Z * var_Z / 100.000

# covert XYZ to RGB

var_R = X * 3.2406 + Y * -1.5372 + Z * -0.4986

var_G = X * -0.9689 + Y * 1.8758 + Z * 0.0415

var_B = X * 0.0557 + Y * -0.2040 + Z * 1.0570

# gamma correction to IEC 61966-2-1 standard

if var_R > 0.0031308: var_R = 1.055 * ( var_R ** ( 1 / 2.400 ) ) - 0.055

else: var_R = 12.92 * var_R

if var_G > 0.0031308: var_G = 1.055 * ( var_G ** ( 1 / 2.400 ) ) - 0.055

else: var_G = 12.92 * var_G

if var_B > 0.0031308: var_B = 1.055 * ( var_B ** ( 1 / 2.400 ) ) - 0.055

else: var_B = 12.92 * var_B

# trim

if (var_R*255) > 255: R = 255

elif (var_R*255) < 0: R = 0

else: R = round(var_R*255)

if (var_G*255) > 255: G = 255

elif (var_G*255) < 0: G = 0

else: G = round(var_G*255)

if (var_B*255) > 255: B = 255

elif (var_B*255) < 0: B = 0

else: B = round(var_B*255)

colours.append([R,G,B])

return numpy.array(colours)

# To call this; specify L, a, b and radius.

colours = LAB2RGB(L = 50, a = 20, b = 20, radius = 60)

**Output from the All-Item analyses**

Here, we present and discuss the output of the model including memory for all four items. For all four items, the between-item model also had a smaller WAIC than the within-item model (Δ = -545.6). Therefore, we present and discuss parameter estimates from this model below, see Table S3 for output from the within-item models.

**Memory performance: Parameter estimates**

For all items, there was ‘anecdotal’ evidence for a main effect of age on the probability of remembering colors (P^M^), as well as a ‘decisive’ verbalization effect (BF_10_ = 6.14 × 10^18^) and an interaction between the two (BF_10_ = 7.28), suggesting that verbalization affected performance differently in the two age groups. Specifically, the younger adults were comparatively more impaired by suppression (see Fig. S3; see also suppression vs. labelling analysis, Age Group × Verbalization BF_10_ = 118.18, Table S2). There was no main effect of age on the probability of continuous representations, but a ‘substantial’ main effect of verbalization (BF_10_ = 9.34), and evidence for an interaction with age (BF_10_ = 5.05). For precision, we observed ‘strong’ evidence for an age effect (BF_10_ = 10.98) and a ‘decisive’ verbalization effect (BF_10_ = 3.25 × 10^5^), but no Age Group × Verbalization interaction, suggesting that verbalization instructions had similar effects on continuous memory precision in both age groups.

**Preventing Labelling (Silence vs. Suppression)**

When modelling performance for all four items in the arrays together, suppression also reduced the probability of remembering (P^M^; BF_10_ = 3.19 × 10^21^). Also, there was some evidence for an interaction with age, such that the younger adults were comparatively more impaired by suppression (BF_10_ = 5.61). Suppression increased the probability of continuous representations (P^O^; BF_10_ = 60.49) in both age groups (see Fig. S1). Hence, when responding to all items, suppression reduced categorical responding in both age groups, not just for the older adults (in contrast to the traditional, first-presented items analysis above). Categorical memory representations in older adults' reduced credibly under suppression (*M* = - 0.71 items), and in younger adults (*M* = - 0.68 items), but there were no credible differences in continuous K in either age group (see Fig. S2). There was also ‘decisive’ evidence that suppression resulted in reduced precision (BF_10_ = 6.06 × 10^4^), and this was not observed differently in the age groups.

**Enforcing Labelling (Silence vs. Labelling)**

Overt labelling also improved memory (P^M^) for both age groups for all items (BF_10_ = 1.19 × 10^11^). There was no evidence of a main effect of labelling on the probability of continuous responding (P^O^), but substantial evidence for an interaction between labelling and age group (BF_10_ = 17.02), indicating that enforced labelling made young adults less likely to have continuous representations but older adults slightly more, compared to silence (see Fig. S1). Compared to spontaneous performance in silence, younger adults' categorical memory representations increased credibly when instructed to label (*M* = 0.49 items), while their continuous K credibly decreased (*M* = - 0.20 items). In contrast, older adults' categorical memory capacity under instructed labelling did not differ credibly from their performance in silence (see Fig. S2), while their continuous capacity increased credibly (*M* = 0.26). There was ‘anecdotal' evidence that instructed labelling increased precision (BF_10_ = 1.33) to equal extents in the age groups.

**The Labelling Benefit (Labelling vs. Suppression)**

***All items.*** Including the subsequent three items in the analysis produced a different pattern. While overt labelling improved memory (P^M^) in both age groups compared to suppression (BF_10_ = 3.01 × 10^38^), it also increased the probability of categorical responding (1 - P^O^, BF_10_ = 350.58), seemingly not to different extents in the age groups (Age Group × Verbalization; BF_10_ = 0.55). Similar to first-presented items, labelling led to credible increases in categorical K compared to suppression for participants of both age groups (younger *M* = 1.18, older *M* = 0.75 items). Surprisingly, the older adults’ continuous K benefitted as well (*M* = 0.19 items), but the younger adults’ did not (see Fig S2). This suggests that labelling benefitted memory performance differently as participants responded to subsequent items. We observed decisive evidence that suppression decreased precision (σ^O^) compared to overt labelling (BF_10_ = 4.68 × 10^8^), to similar extents in both age groups (Age Group × Verbalization; BF_10_ = .26).

**Self-reported verbal labeling: The effect on performance**

As an exploratory analysis suggested by a reviewer, we tested whether participants who reported using a verbal strategy in the silent condition were more impaired by suppression, and/or benefited less from overt labeling. For younger adults, we found inconclusive evidence that labelers differed in overall memory performance (P^M^) from non-labelers when comparing the labeling and silence conditions (BF_10_ = 2.10), and a labeling group × verbalisation interaction (BF_10_ = 1.54).There was no clear evidence that the number of continuous/categorical representations differed between labellers and non-labelers (BF_10_ = 0.15), and no interaction with labeling instruction (BF_10_ = 0.015). When comparing younger adult labelers and non-labelers in the suppression and silence conditions, we observed no clear evidence that self-reported labelers performed differently from non-labelers (BF_10_ = 0.26), and no evidence for a group type × verbalisation instruction interaction (BF_10_ = 0.026). Similarly, there was no clear evidence that the number of continuous/categorical representations changed by labeling-group (BF_10_ = 0.19, interaction; BF_10_ = 0.23).

For the older adults, there was no clear evidence that self-reported labelers differed in overall memory performance (P^M^) from non-labelers, when comparing the labeling and silence conditions (BF_10_ = 0.14), and we observed evidence against a labeling-group × verbalization condition interaction (BF_10_ = 0.0063). We observed no clear evidence that the number of continuous/categorical representations changed by group type (BF_10_ = 0.17) nor for a labeling-group × verbalization interaction (BF_10_= 0.14). Finally, there was no clear evidence that self-reported labelers performed differently non-labelers when comparing the suppression and silence conditions (P^M^; BF_10_ = 0.14), and no evidence for a labeling-group × verbalization interaction (BF_10_ = 0.018). There was also no clear evidence that the number of continuous/categorical representations changed by group type (BF_10_ = 0.20, labeling-group × verbalization interaction; BF_10_ = 0.25). However, the number of self-reported non-labelers was relatively small (7 younger adults, 9 older adults).

**The Labelling Benefit despite Interference and Delay**

In the paper, we focused on the effect of labelling in the ‘traditional' analysis: memory for the first-presented item only. This is standard practice because memory for subsequent items is "tainted" by interference from previous responding. Arguably, however, in real-world contexts maintaining representations despite interference is common, and interchangeable use of visual/verbal representations is likely involved in this process. For instance, some suggest that perceptual memories are lost via 'sudden death' rather than gradual decay (Zhang & Luck, 2009), whereas memories with verbal labels are more robust (Donkin, Nosofsky, Gold, & Shiffrin, 2015). For all four items, labelling (compared to suppression) increased categorical representations but not continuous representations in the younger adults. This differed from Souza and Skóra’s (2017) results; they found categorical and continuous benefits of labelling for one item and all items alike. However, our longer presentation time (930 compared to 250 ms; used to ensure older adults would be able to perceive and label colors) may have induced these differences by making our task easier. Visual traces were likely much stronger, even under suppression and response interference, for our participants.

Surprisingly, for all four items, in older adults both categorical and continuous representations increased with overt labelling compared to suppression. Compared to silence, labelling only boosted continuous representations. This supports the idea that older adults maximized categorical representations by sub-vocally rehearsing in silence but that saying labels out loud – thus producing auditory traces – protected their visual (i.e. continuous) representations from the response interference associated with responding to all four items, or the delay imposed as they provided responses one by one. Indeed, the memory decay with time from the initial presentation to giving the final response was likely exacerbated in older adults due to slower processing speed (Brown, Brockmole, Gow, & Deary, 2012). Also, the appearance of the color-wheel as a response device likely interferes with the original visual trace (Donkin, et al., 2015). As participants look around the wheel and provide their responses, this interference might affect older adults differently if they are more susceptible to distraction (supported by, e.g., Gazzaley, Cooney, Rissman, & D'Esposito, 2005), and overt labelling might have helped reduce the decline of perceptual traces despite such interference. These results suggested that labels can play different roles when providing multiple memory responses in participants of different age groups.

**References**

Brown, L. A., Brockmole, J. R., Gow, A. J., & Deary, I. J. (2012). Processing speed and visuospatial executive function predict visual working memory ability in older adults. *Experimental aging research*, *38*(1), 1-19.

Donkin, C., Nosofsky, R., Gold, J., & Shiffrin, R. (2015). Verbal labeling, gradual decay, and sudden death in visual short-term memory. *Psychonomic Bulletin & Review*, *22*(1), 170-178.

Gazzaley, A., Cooney, J. W., Rissman, J., & D'esposito, M. (2005). Top-down suppression deficit underlies working memory impairment in normal aging. *Nature neuroscience*, *8*(10), 1298.

Souza, A. S., & Skóra, Z. (2017). The interplay of language and visual perception in working memory. *Cognition*, *166*, 277-297.

Zhang, W., & Luck, S. J. (2009). Sudden death and gradual decay in visual working memory. *Psychological science*, *20*(4), 423-428.
